# Dynamic evolution in the key honey bee pathogen deformed wing virus: Novel insights into virulence and competition using reverse genetics

**DOI:** 10.1371/journal.pbio.3000502

**Published:** 2019-10-10

**Authors:** Eugene V. Ryabov, Anna K. Childers, Dawn Lopez, Kyle Grubbs, Francisco Posada-Florez, Daniel Weaver, William Girten, Dennis vanEngelsdorp, Yanping Chen, Jay D. Evans

**Affiliations:** 1 Bee Research Laboratory, Beltsville Agricultural Research Center, Agricultural Research Service, USDA, Beltsville, Maryland, United States of America; 2 Beeweaver Apiaries, Navasota, Texas, United States of America; 3 Department of Chemistry, Fort Lewis College, Durango, Colorado, United States of America; 4 Department of Entomology, University of Maryland, College Park, Maryland, United States of America; The Pennsylvania State University, UNITED STATES

## Abstract

The impacts of invertebrate RNA virus population dynamics on virulence and infection outcomes are poorly understood. Deformed wing virus (DWV), the main viral pathogen of honey bees, negatively impacts bee health, which can lead to colony death. Despite previous reports on the reduction of DWV diversity following the arrival of the parasitic mite *Varroa destructor*, the key DWV vector, we found high genetic diversity of DWV in infested United States honey bee colonies. Phylogenetic analysis showed that divergent US DWV genotypes are of monophyletic origin and were likely generated as a result of diversification after a genetic bottleneck. To investigate the population dynamics of this divergent DWV, we designed a series of novel infectious cDNA clones corresponding to coexisting DWV genotypes, thereby devising a reverse-genetics system for an invertebrate RNA virus quasispecies. Equal replication rates were observed for all clone-derived DWV variants in single infections. Surprisingly, individual clones replicated to the same high levels as their mixtures and even the parental highly diverse natural DWV population, suggesting that complementation between genotypes was not required to replicate to high levels. Mixed clone–derived infections showed a lack of strong competitive exclusion, suggesting that the DWV genotypes were adapted to coexist. Mutational and recombination events were observed across clone progeny, providing new insights into the forces that drive and constrain virus diversification. Accordingly, our results suggest that *Varroa* influences DWV dynamics by causing an initial selective sweep, which is followed by virus diversification fueled by negative frequency-dependent selection for new genotypes. We suggest that this selection might reflect the ability of rare lineages to evade host defenses, specifically antiviral RNA interference (RNAi). In support of this hypothesis, we show that RNAi induced against one DWV strain is less effective against an alternate strain from the same population.

## Abstract 2

Deformed wing virus, a key pathogen of honey bees, shows rapid diversification after genetic bottlenecks; a novel reverse-genetic system provides insights into the forces that shape virus diversity, suggesting that virus quasi-species diversification may be driven by selection of genotypes capable of evading host RNAi defences.

## Introduction

**Fig 1:**
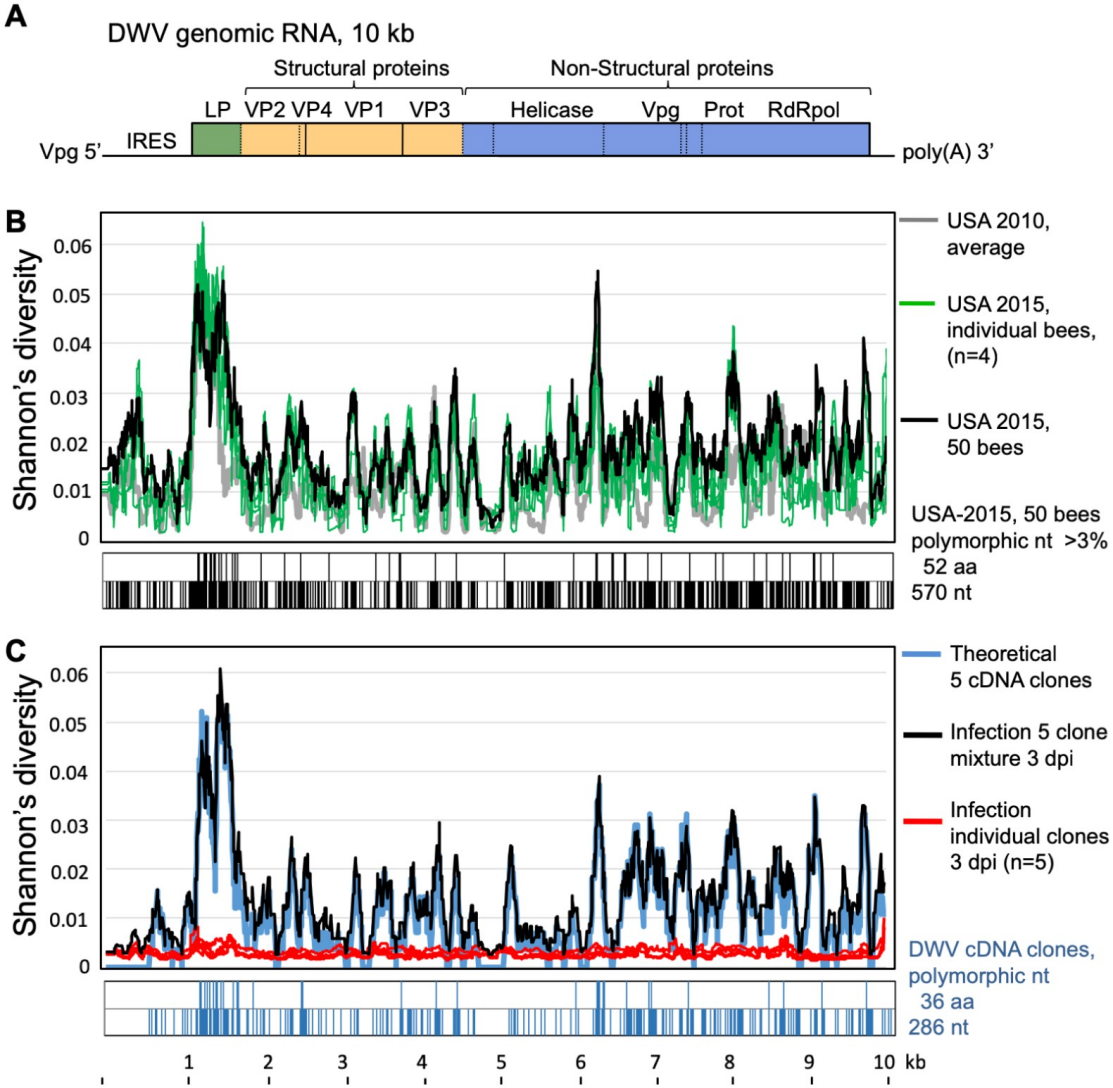
Polymorphisms in natural and clone-derived DWV. (A) Reference map of DWV genomic RNA. (B,C) Shannon's diversity profiles for DWV (averaged for 100 nt) for (B) 2010 bees (gray line), 2015 individual honey bees (green lines), and a colony sample (black line); (C) individual bees infected with cDNA clone-derived DWV isolates—red lines, single infections; black line, mixed infection of five clones; blue line, theoretical profile for five clone mixture. Distributions of polymorphic nucleotides showing an alternate allele exceeding 3% in frequency and the resulting amino acid changes are shown below the diversity graphs. NGS libraries’ IDs (S1 Table) for the groups are shown in brackets: “USA 2010, average” (B2_PBS, B5_PBS, G2_PBS, G5_PBS, B2_Hem, B5_Hem, G2_Hem, G5_Hem), “USA 2015, individual bees” (V52_Pupa15, V56_Pupa15, V57_Pupa15, V62_Pupa15), “USA 2015, 50 bees” (V99_VIROCT15), “Infection 5 clone mixture 3 dpi” (V112_ABCDE_3), and “Infection individual clones 3 dpi” (V22_304TR, V25_306TR, V16_422TR, V19_702TR, V21_703TR). dpi, days postinoculation; DWV, deformed wing virus; ID, identifier; IRES, internal ribosome entry site; LP, leader protein; NGS, next-generation sequencing; Prot, viral protease; RdRpol, RNA-dependent RNA polymerase; VP, structural viral protein; Vpg, genome-linked protein.

Population fluctuations affect virulence of invertebrate RNA viruses including the positve-strand RNA deformed wing virus (DWV; Fig 1A), a major pathogen of honey bees. DWV includes three master variants, the most widespread being DWV-A, followed by *Varroa destructor* virus 1 (VDV1 or DWV-B), and infrequently, DWV-C [1–5]. Prevalance of DWV, tightly linked with its invasive vector, the ectoparasitic mite *V*. *destructor* [6], increases honey bee colony mortality [7,8], thereby threatening food security worldwide by affecting crop pollination [9]. Prior to *Varroa* invasion, DWV was transmitted vertically and orally [2,5], and virus infections were likely to have low titers and be nonsymptomatic, similar to infection dynamics observed in the *Varroa*-free Hawaiian Islands [10]. Indeed, a phylogeographic study confirmed that the DWV pandemic and increase in virus virulence coincided with the global spread of the *Varroa* mite [11]. This suggests that DWV populations in *Varroa*-free and *Varroa*-infested colonies are genetically distinct and that mite vectoring drives evolutionary changes in DWV. However, it is not clear which genetic changes make *Varroa*-transmitted DWV more virulent. Previous reports suggested that the VDV1-DWV recombinants detected in the United Kingdom and France [12–14] were associated with mite transmission, although similar recombinants were also present in UK *Varroa*-free honey bees [13]. The most striking difference found in DWV populations associated with mite transmission was a greatly reduced genetic diversity. This was observed in Hawaii, where a sudden drop of DWV diversity occurred following mite invasion [10]. Additionally, in the UK, nearly clonal diversity of DWV-like viruses was reported in individual symptomatic *Varroa*-infested honey bees, contrasting with high viral variability in asymptomatic bees with low DWV levels [13]. Interestingly, the arrival of *Varroa* in Hawaii also preceded a significant reduction of DWV diversity in the wasp *Vespula pensylvanica*, a honey bee predator [15]. Together, these reports suggested that low genetic diversity could be a universal feature of the mite-transmitted DWV. Surprisingly, our analysis of the *Varroa*-associated DWV population currently circulating in the mainland US indicated high genetic diversity of DWV. To investigate the evolutionary dynamics of DWV, we designed a reverse-genetics system for a virulent DWV population by cloning variants coexisting in a typical US DWV-A quasispecies [16], the first of this kind for an RNA invertebrate virus. Using this system, we demonstrated that replication levels of individual US DWV genotypes were equivalent to divergent wild-type DWV, without strong competitive exclusion in mixed infections. Recombination events between DWV isolates were widespread, contributing to virus diversification. We propose a model of DWV dynamics, potentially consistent with punctuated evolution [17], whereby introduction of *Varroa*-selected genotypes causes a selective sweep, after which diversification via negative frequency-dependent selection results in high genetic heterogeneity, potentially benefiting the virus’s ability to escape genotype-specific antiviral defenses.

## Results

### High genetic diversity of DWV in US *Varroa*-infested honey bee colonies reflects postbottleneck expansion

**Fig 2:**
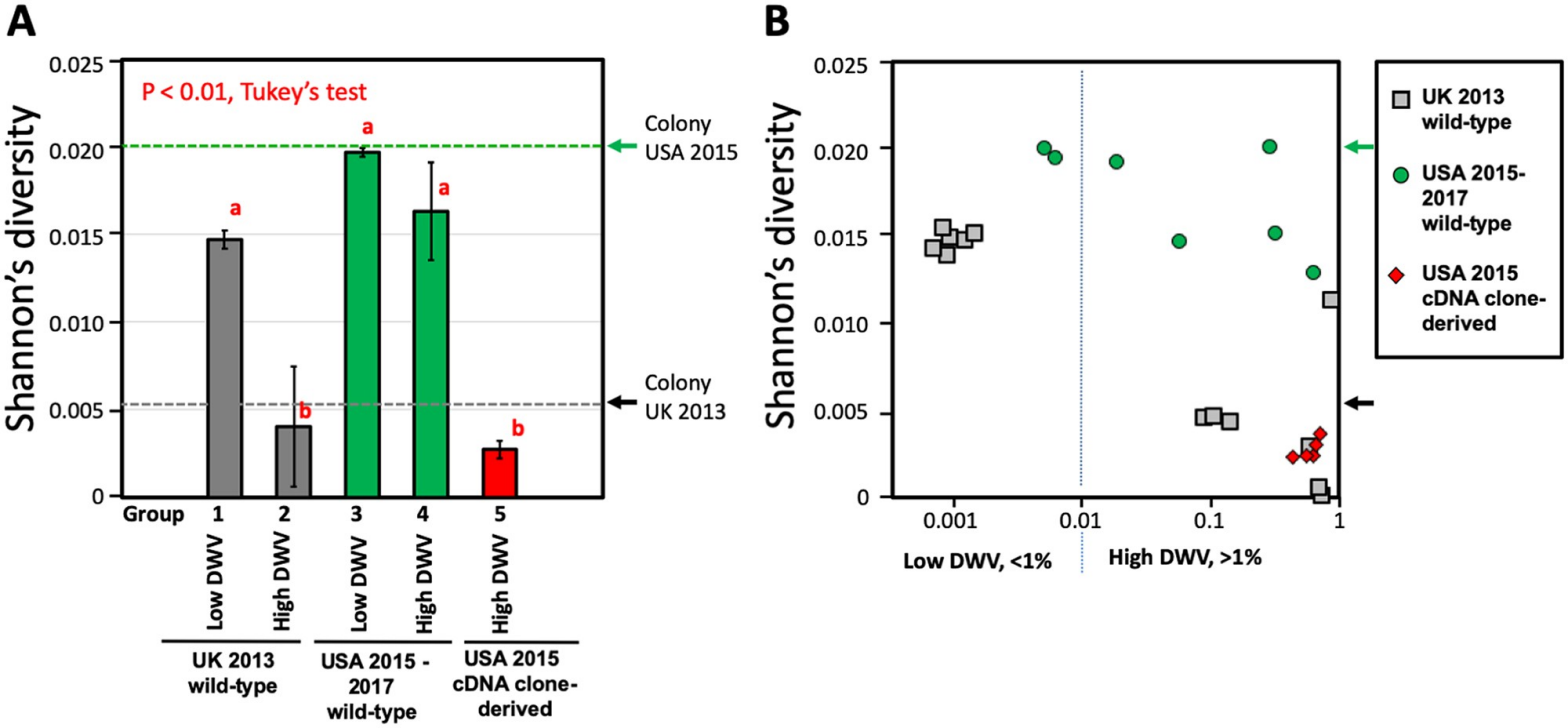
The connection between virus levels in the US and UK honey bees. (A) The columns show the average Shannon's diversity index for the NS region of DWV genomic RNA for individual honey bees from the UK (UK 2013 wild-type) and the USA (USA 2015–2017 wild-type) with low DWV or high DWV levels (less than 1% or more than 1% of DWV reads in NGS library, correspondingly) and honey bees infected with the clone-derived US DWV variants. Error bars indicate standard deviation. The groups with significantly different (*P* < 0.01) Shannon’s diversity index values are indicated above the bars with different letters. (B) Average Shannon's diversity index for the NS region plotted against the proportion of DWV in the NGS library. (A, B) Arrows at the right side of the graphs indicate Shannon’s diversity index values of the colony-level DWV populations in the UK and the USA were calculated for the NGS libraries for virus preparations from 50 bees. The DWV NS region corresponds to the positions 5,500–9,847 of the US DWV isolate DWV-304 (GenBank accession number MG831200). NGS libraries’ IDs (S2 Table) for the groups are shown in brackets: group 1 (UK -A1, UK-A3, UK-B1, UK-B2, UK-F3, UK-F7), group 2 (UK-E7, UK-E8, UK-E10, UK-E11, UK-INJ4, UK-INJ5, UK-INJ6), group 3 (V113_WT15_0, V31_PBS), group 4 (V52_Pupa15, V56_Pupa15, V57_Pupa15, V62_Pupa15, V114_WT15_3), group 5 (V22_304TR, V25_306TR, V16_422TR, V19_702TR, V21_703TR), colony UK 2013 (UK-VIR2), and colony USA 2015 (V99_VIROCT15). The libraries’ statistics is provided in S2 Table, NGS coverage and Shannon’s diversity profiles for the analyzed region are shown in S1 Fig, and numerical values underlying the summary graphs are provided in S1 Data. DWV, deformed wing virus; ID, identifier; NGS, next-generation sequencing; NS, nonstructural.

Honey bee viromes comprehensively characterized by next-generation sequencing (NGS) (S1 Table) had surprisingly high DWV genetic diversity in US *Varroa*-infested colonies compared with prior population surveys of the *Varroa*-associated DWV carried out in the UK in 2013 [13] (Fig 2, S2 Table, and S1 Fig). In particular, the levels of DWV genetic diversity in individual US honey bees with high DWV levels (Fig 2A, group 4) were significantly higher than in overtly infected UK bees with high DWV levels [13] (Fig 2A, group 2), contradicting the previously reported nearly clonal nature of DWV in *Varroa*-infested colonies in the Hawaiian Islands [10] and UK [13]. In fact, US honey bees with both high and low DWV levels (Fig 2A, groups 3 and 4) showed virus diversity levels similar to those observed in covertly infected UK bees with low DWV levels (Fig 2A, group 1). Significantly higher virus diversity in US honey bees compared with UK bees was also observed at the colony level when the pool of 50 bees was analyzed by NGS. Whereas the UK colony-level DWV population from a Warwickshire *Varroa*-infested colony sourced in July 2013 was nearly clonal (Fig 2A and 2B; “Colony UK 2013,” black arrows), the diversity of DWV in the US *Varroa*-infested colony sourced in October 2015 in Maryland (Fig 2A and 2B; “Colony USA 2015,” green arrow) was even higher than in the UK bees with low DWV levels, suggesting that DWV populations in the US have more genetic variants.

**Fig 3:**
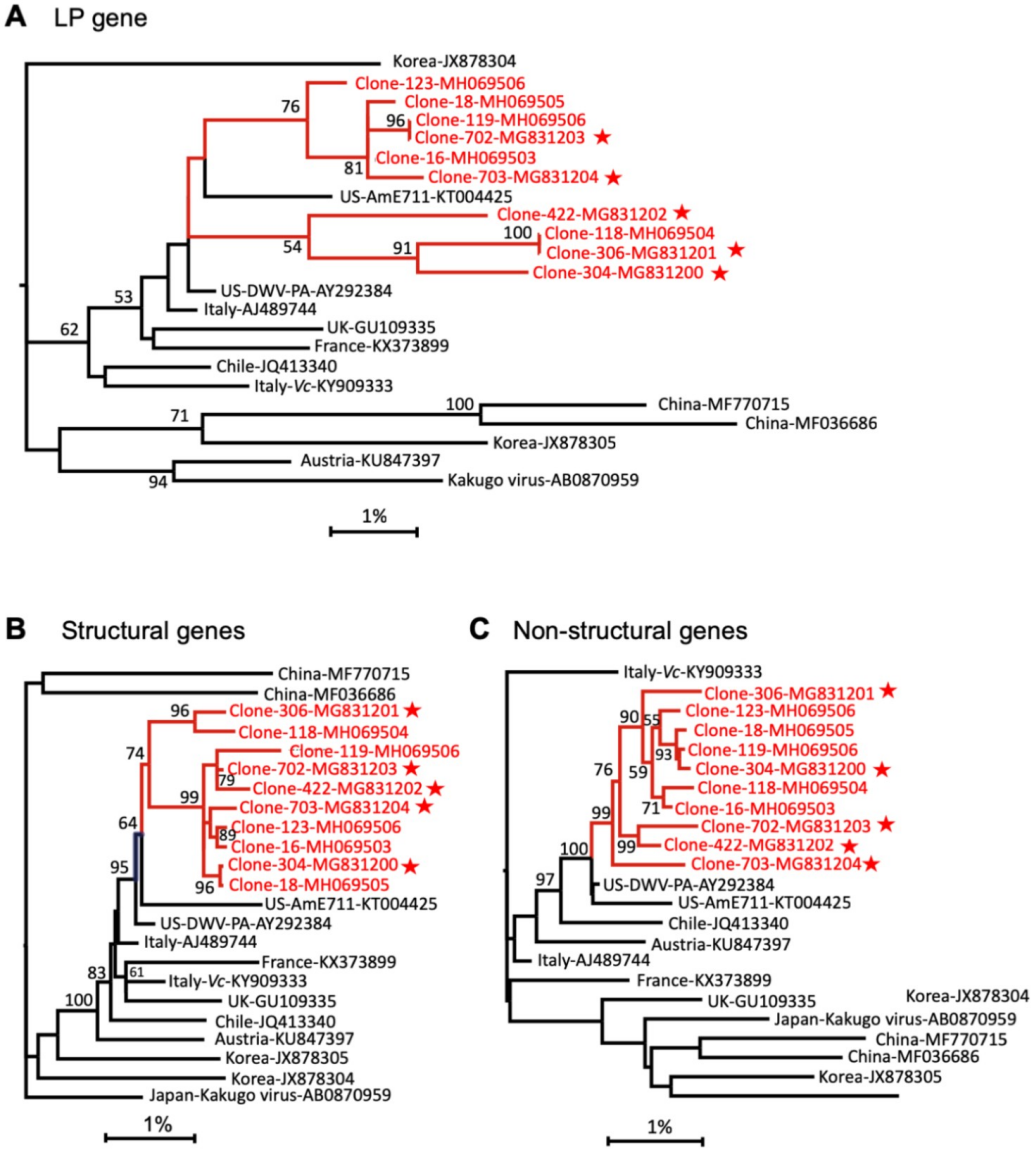
Phylogeny of genomic segments of DWV RNAs. Maximum likelihood phylogenetic trees were generated for the sequences coding for the (A) LP (positions 1.1–1.7 kb), (B) structural proteins (positions 1.7–4.3 kb), and (C) major nonstructural proteins (positions 4.3–10.1 kb). Nodes connecting the cloned sequences (“Clone-“), are shown in red; stars indicate DWV genotypes with tested infectivity. Bootstrap values for 1,000 replicates are shown for groups with more than 50% bootstrap support. Scale shows genetic distance (%). DWV, deformed wing virus; LP, leader protein.

Indeed, analysis of the divergent position distribution in a virulent DWV population from a US colony that collapsed 3 months after sampling showed that it had 5.6% polymorphic nucleotides across the viral genome, in which the proportion of the alternate nucleotide exceeded 3%, generating 1.8% amino acid substitutions (Fig 1B, “USA 2015, 50 bees”; S1 Table, V99). NGS analysis also showed that this collapsed honey bee colony included exclusively DWV genotypes of type A (DWV-A), most closely related to US isolates DWV-PA [1] and DWV-Ame711 [18], and did not harbor VDV1 type, which has spread rapidly in the US in the last decade [19]. DWV polymorphism levels and distribution of diversity in individual honey bee pupae (Fig 1B, “USA 2015, individual bees”) closely matched this single-colony sample (Fig 1B, “Virus-USA 2015”), with diversity profile Pearson’s correlation coefficients ranging from 0.7453 to 0.9549 (S3 Table). Moreover, bees collected in 2010 in two apiaries in Texas and Maryland had DWV diversity profiles similar to the 2015 Maryland samples (Fig 1B, “USA 2010, average”; 2010 and 2015 Pearson’s correlation coefficients ranged from 0.5287 to 0.7432, S3 Table). Given the phylogenetic relatedness between the DWV consensus sequences from 2010 and 2015 (Fig 3 and S2A Fig, sequences labeled with suffix “-Cons”), a stable divergent DWV population, like the one we report in this study, is the most widespread, or at least very common, in *Varroa*-infested bees across the mainland US and has existed since at least 2010.

Phylogenetic analysis of the nearly full-length cloned DWV genomes (9.7-kb sections containing the full protein-coding sequences) produced for this study from a *Varroa*-infested Maryland colony (S2 Fig, sequences labeled with prefix “Clone-”) revealed that they formed a cluster that also included the source colony’s NGS consensus (S1 Table, NGS library V99_VIROCT15), consensus sequences from individual bees from the same apiary, and the US 2010 bees from Maryland and Texas (S2 Fig, sequences labeled with suffix “-Cons”). This clade, which had 98% bootstrap support, was rooted in a branch with all complete DWV-A genomes from the US and the NGS-derived DWV sequences for 2010 and 2017 US bees from this study, which had 100% bootstrap support (S2A Fig). All cloned viral genomes belonged to DWV-A type (S2B Fig), which was in good agreement with NGS analysis of the source Maryland colony (S1 Table, NGS library V99_VIROCT15). Specific genome sections revealed similar phylogenetic relationships (Fig 3), further corroborating that the US DWV-A population was generated following a strong bottleneck event and subsequent diversification from a single, or closely related, strain(s) from Europe.

### Design of the reverse-genetics system for a virulent US DWV population

**Fig 4:**
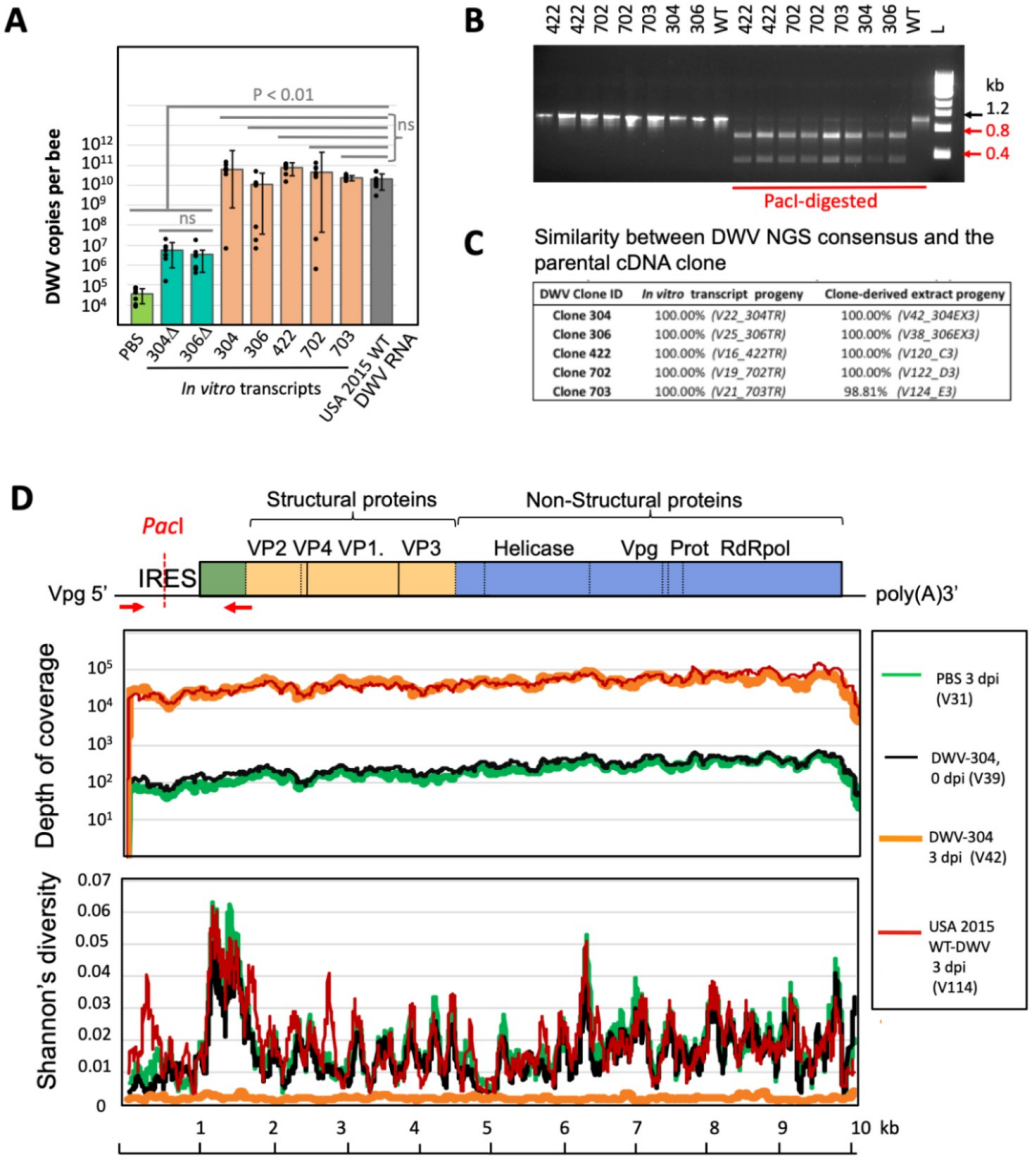
Infectivity of clone-derived DWV isolates. (A) Replication of DWV in honey bee pupae injected with 5 μg of in vitro RNA transcripts from full-length infectious DWV cDNA clones (304, 306, 422, 702, and 703), DWV cDNA constructs with a deletion of essential replication genes (Δ304, Δ306), 5 μg of WT DWV RNA (USA 2015 WT DWV RNA), or buffer control (PBS). The injected pupae, *n* = 6 per each group, were sampled at 3 dpi, and the DWV loads per individual pupa were quantified by qRT-PCR. Black dots indicate DWV load in individual honey bees. The columns show the average DWV copy numbers for each treatment ± SD. Statistically significant (*P* < 0.01) and ns differences between treatments groups are indicated above the bars. Numerical values underlying the summary graphs are provided in S1 Data. (B) Presence of the introduced *Pac*I restriction site in the progeny of the clone-derived DWV genomes in the in vitro transcript-injected pupae at 3 dpi. The RT-PCR DNA products corresponding to the 5′ terminal part of the DWV RNA, untreated or digested with *Pac*I, were separated by agarose gel electrophoresis. (C) NGS analysis of the cDNA clone-derived DWV progeny from the in vitro transcript-injected and clone-derived extract-injected honey bee pupae. Shown is the percentage of similarity between the DWV consensus sequences produced from NGS libraries (sample ID indicated in brackets) compared with their respective parental DWV cDNA sequences. (D) NGS analysis of the pupae infected with divergent wild-type DWV and clone-derived DWV (clone 304) immediately after injection (0 dpi) and at 3 dpi. NGS library ID is contained within parentheses. dpi, days postinoculation; DWV, deformed wing virus; ID, identifier; IRES, internal ribosome entry site; NGS, next-generation sequencing; ns, nonsignificant; Prot, viral protease; qRT-PCR, quantitative RT-PCR; RdRpol, RNA-dependent RNA polymerase; RT-PCR, reverse-transcription PCR; VP, structural viral protein; Vpg, genome-linked protein; WT, wild-type.

Stable coexistence of multiple variants within a virulent DWV population prompted questions about interactions between virus genotypes and driving forces maintaining high diversity. We used a molecular approach to investigate these interactions, which involved producing a series of full-length infectious cDNA clones of genomes of distinct isolates coexisting in a DWV population from a single colony (Fig 3 and S2 Fig, sequences labeled with a star). Together, these clones captured a significant proportion of the genetic diversity present in a typical US DWV-A population (Fig 1C, blue line). The colony used for virus preparation was *Varroa* infested, contained honey bees showing wing deformities consistent with high DWV levels [2,6], and did not survive the following winter season, indicating that it harbored a virulent DWV strain typical of declining US colonies. The selected clones (*n* = 5) and parental population (“USA-2015, 50 bees”) had highly similar distributions of divergent nucleotides (*n* = 286) and amino acids (*n* = 36) (Fig 1C and 1B). Shannon’s diversity profile for equal proportions of each clone (Fig 1C, blue line) was positively correlated (Pearson’s R = 0.804; S3 Table) with that of the parental DWV population (Fig 1B, black line). When injected into honey bee pupae, full-length DWV in vitro RNA transcripts generated from the cDNA clones were infectious. The clone-derived DWV isolates replicated to similarly high levels, about 10^10^–10^11^ genome copies per bee, as wild-type RNA of the same DWV preparation used to design the clones. In addition, typical 30-nm DWV virus particles were observed in filtered tissue extracts from the in vitro transcript-injected pupae (S3 Fig) 4 days postinoculation (dpi). Significantly lower (Wilcoxon rank sum test, *P* < 0.01) virus levels were observed in pupae injected with PBS or with the mutant transcripts 304Δ and 306Δ, in which essential viral genes required for replication were deleted (Fig 4A). Because DWV is widespread and present at low levels, about 10^5^–10^7^ copies per insect in virtually all honey bees, including *Varroa* free [5,13], this virus load was detected by quantitative reverse-transcription PCR (qRT-PCR) even in control samples. Therefore, it was important to determine that the virus that replicated to high levels in the DWV transcript-injected honey bees was indeed clone derived. The identity of clone-derived DWV progeny in the injected pupae was confirmed both by using the unique restriction site introduced into the clones (Fig 4B) and by NGS analysis (S1 Table), which showed that the consensus nucleotides of the clone DWV sequences were identical to their respective parental cloned cDNA (Fig 4C), while retaining low diversity (Fig 1C, red lines; Fig 2, group 5—“USA 2015 cDNA clone-derived USA-2015”; S1 Table), thereby proving that the cloned DWV isolates were infectious.

**Fig 5:**
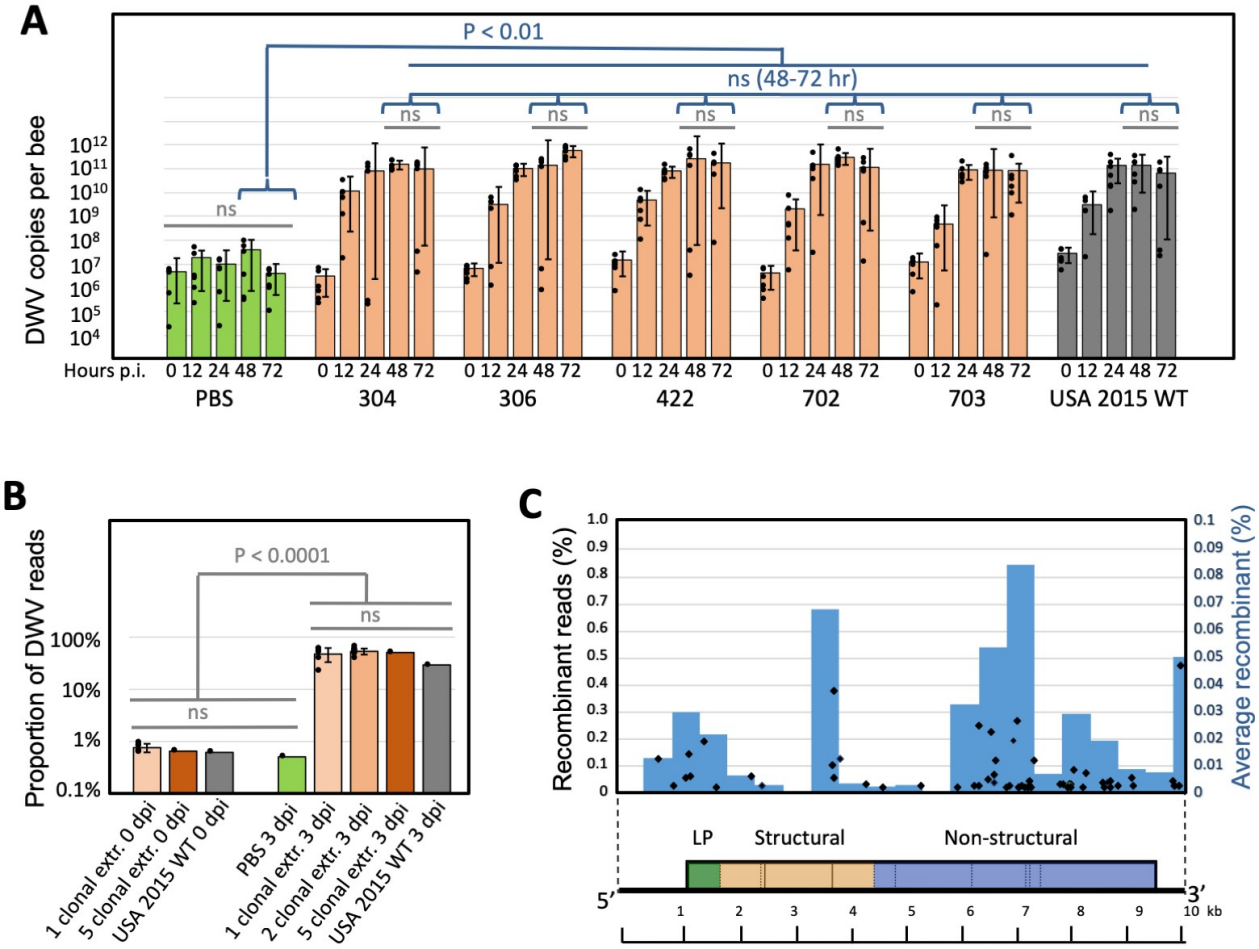
Replication dynamics and interaction between the clone-derived DWV isolates. (A) Average DWV RNA copies per honey bee pupa ± SD. Individual pupae copy numbers indicated by black dots. (B) Proportion of DWV reads in NGS libraries for individual, mixed, and control infections. Significant and ns differences are indicated. (C) Recombination breakpoints generated in mixed clone-derived infections. Black diamonds show the proportion of recombinant reads in individual mixed infections (left y-axis). Blue bars show average proportion of recombinant reads in a genome section (right y-axis). DWV genetic map is shown below; numerical values underlying the summary graphs are provided in S1 Data. dpi, days postinjection; DWV, deformed wing virus; extr, virus extract; LP, leader protein; NGS, next-generation sequencing; ns, nonsignificant; p.i. postinjection; WT, wild-type.

We then investigated replication dynamics of the recovered clone-derived virus isolates. After 48 and 72 hours, DWV levels were the same in pupae injected with 10^7^ genome copies of either clone-derived or wild-type DWV (Fig 5A), further demonstrating that individual DWV genomes replicate to the same level as the parental divergent DWV population (Fig 5A, Wilcoxon rank sum test, *P* > 0.01). Consensus DWV sequences at 3 dpi were identical to their corresponding cDNA clones in all but one case, likely because of a background DWV infection in the injected pupae (Fig 4C). DWV levels at 3 dpi were significantly higher (Fig 5B, Wilcoxon rank sum test, *P* < 0.0001) than 0-dpi pupae sampled immediately after injection with the virus, which had the same levels as PBS-injected controls (Fig 5B). The recipient honey bee pupae had low-titer background infection, which caused DWV diversity to be high in a DWV-304 0-dpi pupa injected with clone-derived DWV (Fig 4D, black lines—V39), but the clone-derived progeny had low, nearly clonal genetic diversity at 3 dpi (Fig 4D, orange lines—V42), indicating that only the hemolymph-injected clone-derived virus replicated to high levels, rather than the highly divergent background, in stark contrast with pupae injected with wild-type DWV (Fig 4D, red lines—V114). Notably, in the honey bee pupa injected with buffer (PBS) control, divergent DWV remained at a low level (Fig 4D, green lines—V31). It could be argued that the mutation rate was too low to detect accumulation of mutant variants within 3 dpi; therefore, future studies may wish to extend the postinjection analysis time frame to allow for direct observation diversification of the clone-derived genotypes.

### Mixed infection of clone-derived DWV genotypes showed a lack of synergistic effects and competitive exclusion

Divergent virus populations might have increased fitness compared with individual genotypes because of complementation between genotypes that could reinforce each other to achieve a higher replication rate [20]. The replication dynamics of the individual clone-derived DWV isolates, their mixtures, and divergent wild-type DWV were compared. When honey bees were infected by hemolymph injection with extracts containing a total of 10^7^ genome equivalents of clone-derived virus particles, there were no significant differences (Wilcoxon rank sum test) between DWV levels in pupae infected with either individual clone-derived isolates, all possible pairwise combinations of these isolates, or a mixture composed of all five isolates (Fig 5B). The DWV Shannon’s diversity profile in pupae injected with the mixture of all five clonal isolates was similar to that predicted, assuming equal proportions of each component (Fig 1C, black and blue lines, respectively; S3 Table, Pearson’s correlation coefficient *P* = 0.9825). Such a tight correlation between theoretical and observed profiles for mixed infections suggests that each of five coinfected genotypes replicated to similar levels in the reassembled population. NGS analysis of infections induced by pairwise clone-derived isolate mixtures (S1 Table) allowed a genome-wide view of strain success via the ratios of alternate nucleotides at the expected divergent positions (S4 Fig). Although some clones showed higher proportions than others, no complete competitive exclusion [21] was observed over 72 hours when resident genotypes of DWV were coinoculated.

### Widespread recombination between the DWV isolates in mixed infections

NGS of the pairwise clone-derived isolate mixtures also allowed us to investigate recombination within DWV populations. Analysis of the changes in the proportion of divergent nucleotides along the DWV genome (S4 Fig, lower panels, orange bars) revealed widespread generation of novel DWV variants as a result of recombination events between the clone-derived genotypes. This was confirmed by an analysis for structural variants that identified recombination breakpoints between clones (S4 Fig, upper panels). Recombination sites were clustered mostly in the regions preceding the leader protein (LP) region, the LP region itself (the main 3′-proximal nonstructural block in the helicase region), and the border of the regions coding for the structural viral protein (VP) 1 and VP3 (Fig 5C). Independent evidence for a high recombination frequency between DWV genotypes came from the phylogenetic analysis of the functional sections of the cloned full-length DWV genomes (Fig 3). It showed different topologies in the phylogenetic trees, supported by high bootstrap values, for the genome sections coding for LP, the structural proteins, and the nonstructural proteins. For example, clone 304 and clone 18 have almost identical structural gene sections (Fig 3B), but their LP gene sections are in different clades (Fig 3A). Similarly, the nonstructural genes of clone 119 and clone 304 are very close (Fig 3C), but their structural genes are in separate branches (Fig 3B). Such differences in tree topologies could be explained by reshuffling of genome sections between members of the DWV population. Recombination events are known to be involved in the generation of novel variants of picorna-like viruses [22]. The fact that recombination events are often located at the borders between the functional blocks reflects the modular nature of the DWV genome. The distribution of recombination sites observed in the mixed infection experiment (Fig 5C and S4 Fig) resembles that found in previous studies. This includes positions flanking the LP region and in the 5′-proximal part of the nonstructural gene block in the recombinants between DWV-A and DWV-B [12–14,23,24], as well as the protease and RNA-dependent RNA polymerase (RdRpol) region of the nonstructural block in the recombinants between Asian DWV-A isolates [25].

### High genetic heterogenicity of DWV populations as an RNA interference evasion mechanism

We found that individual DWV clones from a virulent population replicated to the same levels as did either mixtures of these clones or the parental divergent population (Fig 5B), indicating the absence of obvious synergetic effects. We suggest that diversification of DWV in honey bees and the maintenance of high genetic diversity in wild-type DWV populations might be driven by host antiviral RNA interference (RNAi) defenses [26] and maintained as a way to evade sequence-specific RNAi similar to West Nile virus (WNV) in mosquitoes and Powassan virus in ticks [27–29]. These studies showed that rare genetic variants of these viruses avoid control by RNAi targeting major variants because of the lack of complementarity between the minor strains and the guiding small interfering RNAs (siRNAs) derived from the major viral strains [27–29]. Analysis of the distribution of polymorphic sites in DWV populations provided several findings in support of this suggestion.

We found that most of the nucleotide changes in sampled DWV populations were silent, with a mean ratio of synonymous (dS) to nonsynonymous (dN) substitutions (dS/dN) in the tested infectious clones reaching 24.7, indicating strong purifying selection (S4 Table). Accumulation of a high number of nucleotide changes throughout DWV genomes, constrained by the need to maintain the same coding capacity, suggests that the virus is forced to explore sequence space to maintain its high diversity. This is consistent with the hypothesis that diversification of DWV RNA is driven mostly by selection of novel sequence variants of DWV RNA capable of evading specific RNAi targeting because of mismatches with siRNAs. Analysis of the NGS libraries confirmed that polymorphic sites are present throughout DWV genomes (Fig 1B), in agreement with previous reports that the entire DWV genome could be targeted by RNAi [13,24] and therefore subjected to diversification. Still, a significantly higher density of polymorphic positions was consistently observed in the LP region and in the 3′-proximal part of DWV coding for the nonstructural block (S5 Fig and S4 Table). Additionally, the cloned infectious DWV isolates showed a subset of codons (*n* = 31) with dS/dN < 1, which are potentially subjected to diversifying positive and/or relaxed selection. Interestingly, 12 of these putative positively selected codons were located in the LP region, a 10-fold higher density than in the rest of the genome (S5 Fig and S4 Table).

**Fig 6:**
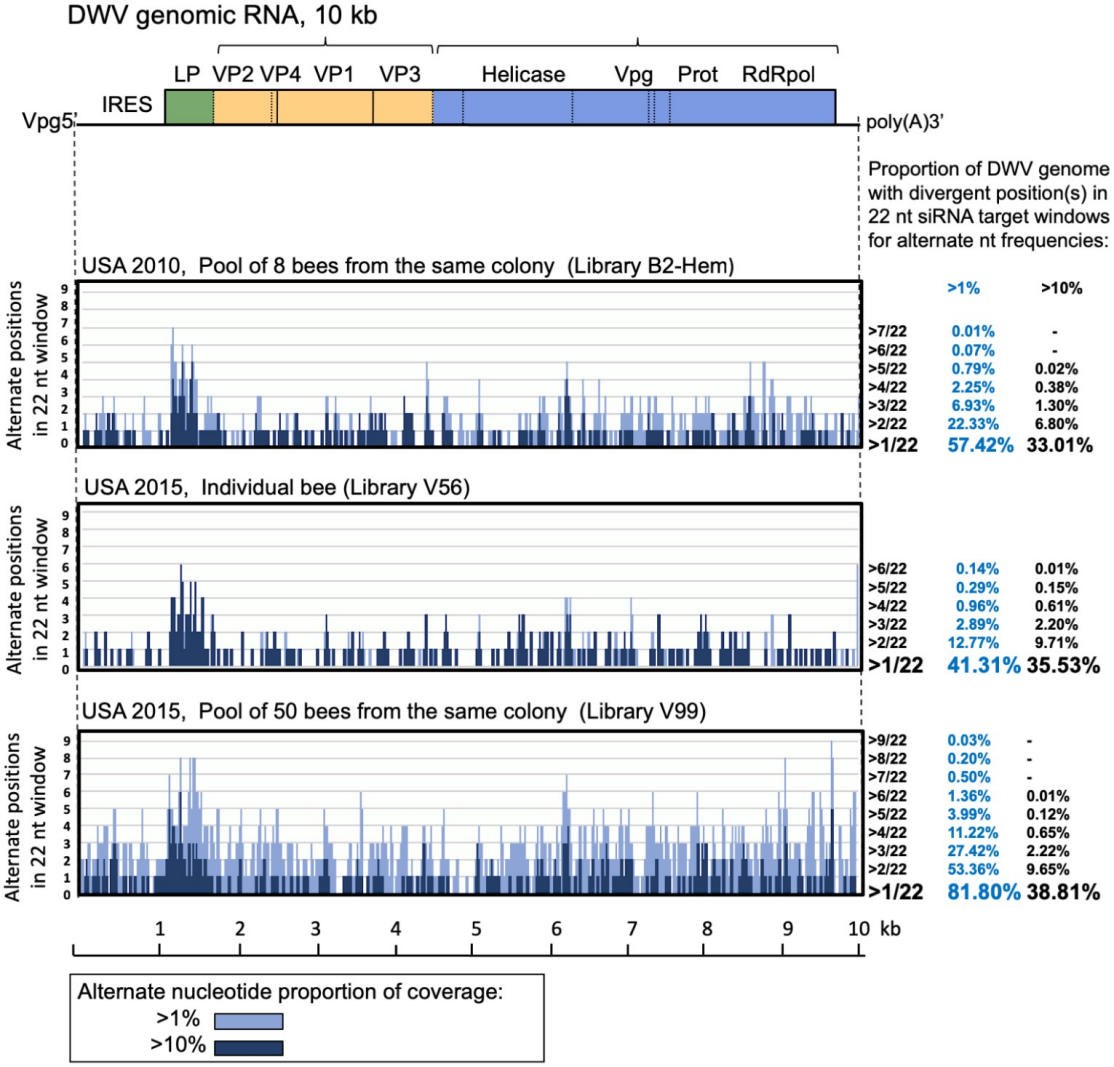
Potential contribution of DWV population diversity to evasion of sequence-specific RNAi targeting. Distribution of the number of alternate positions in 22-nt-long potential siRNA target windows for the polymorphic sites with more than 1% (light blue graphs) and 10% (dark blue graphs) of NGS coverage. A DWV genetic map is shown above the graphs. Summaries of the DWV genome coverage (%) with different number of mismatches in a 22-nt sliding window for each of the NGS libraries is shown at the right of the graph. DWV, deformed wing virus; IRES, internal ribosome entry site; LP, leader protein; NGS, next-generation sequencing; Prot, viral protease; RdRpol, RNA-dependent RNA polymerase; RNAi, RNA interference; small interfering RNA; VP, structural viral protein.

To estimate the potential of US DWV populations to evade specific RNAi established to a single given DWV strain, we analyzed DWV NGS libraries from a typical colony, a single honey bee sampled in 2015, and a pool of eight honey bees sampled in 2010 (S1 Table; Libraries B2_PBS, V57, and V99) for sequence variants capable of avoiding specific RNAi targeting by recording the number of alternate nucleotides (occurring above 1% and 10% levels of the read coverage) in a sliding 22-nt window (Fig 6)—i.e., the size of a potential siRNA target in the honey bee [13,24]. Within the colony-level DWV population, most of the genome, 81.80%, showed at least one sequence variant present at a frequency of 1% or higher, and 38.81% of the genome showed variants present at 10% or higher. Notably, the polymorphic 22-nt windows covered a significant proportion of the viral genome in the DWV populations from the individual bee sampled in 2015, with 35.53% and 41.31% of genomic windows showing sequence variants present at 1% or higher and 10% or higher, respectively. Similarly, the DWV population from a pool of 2010 US honey bees showed 57.42% and 33.01% divergent coverage. Clearly, divergent DWV populations harbor a pool of readily available genetic variants, some of which may possess reduced vulnerability to specific RNAi. Therefore, we propose that widespread recombination events between divergent coexisting DWV genotypes in a population contribute to the generation of variants less effectively targeted by established RNAi, thereby helping DWV to evade this sequence-specific antivirus response.

### Natural differences between DWV genotypes might significantly affect efficiency of antiviral RNAi targeting

**Fig 7:**
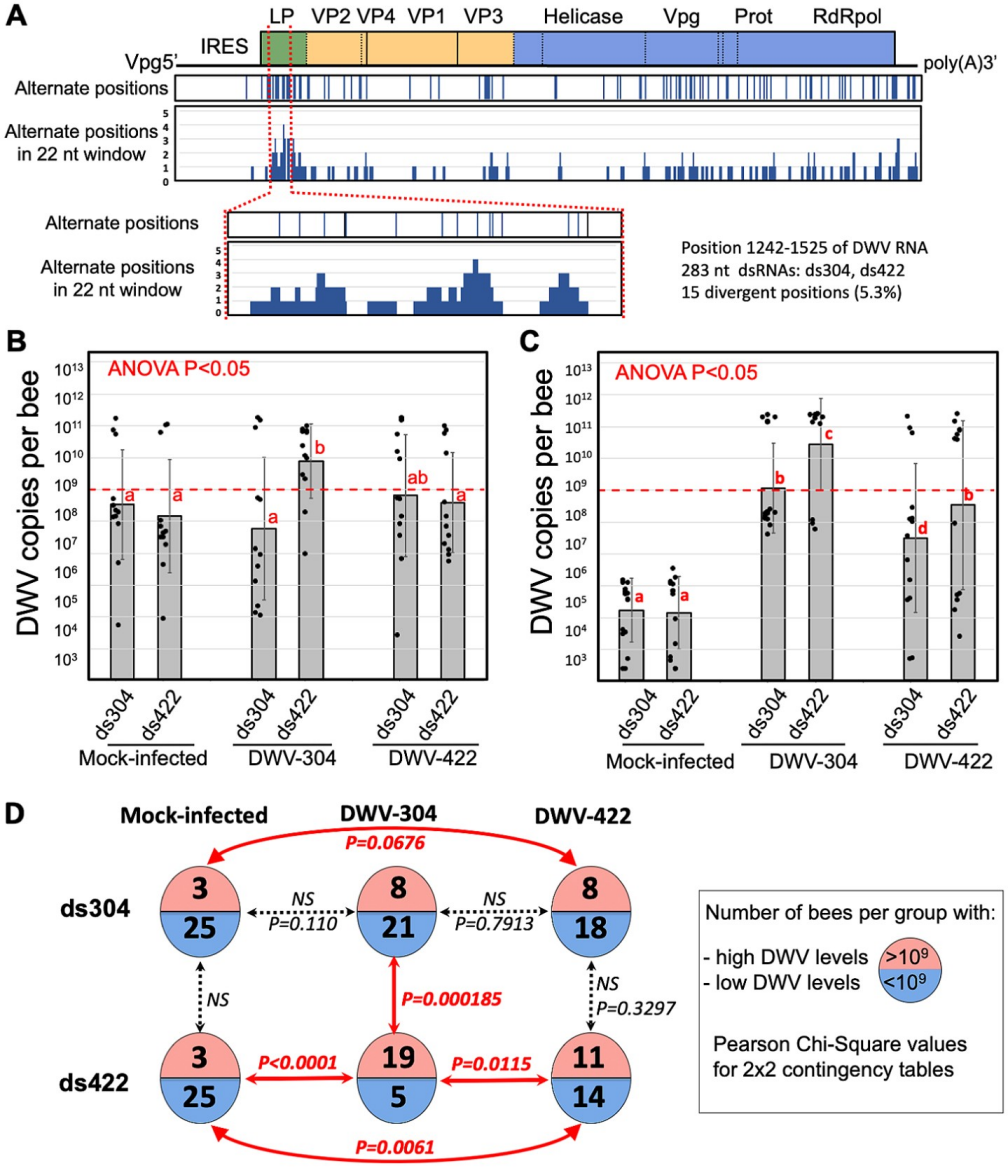
Impact of specificity of RNAi targeting on outcome of DWV infections. (A) Design of the 283-nt dsRNA specific to the DWV isolates 304 and 422 (ds304 and ds422) corresponding to the regions 1,242–1,524 of DWV genome (GenBank accession MG831200). (B, C) DWV loads in the individual adult honey bees 7 days postfeeding with 10^8^ clone-derived virus particles and 1 μg of the DWV-304- or DWV-422-specific dsRNA. Columns show average copy number per treatment groups ± SD; black dots indicate DWV copy numbers in individual honey bees. The groups with significantly different (*P* < 0.01, ANOVA) DWV copy numbers are indicated above the bars with different letters. The red lines indicate the threshold level of 10^9^ DWV genome copies per honey bee, which was used to categorize DWV levels as low or high. Numerical values underlying the summary graphs are provided in S1 Data. (D) Analysis of the DWV infection outcomes in two oral inoculation experiments with the strain-specific dsRNAs. Circles indicate experimental groups; numbers of the bees in each group with high (>10^9^ copies) or low DWV (below 10^9^ DWV copies) levels of DWV are shown on the top or bottom of the circles. A 2 × 2 contingency table analysis was used to test significance of the differences in proportions of the bees with low or high DWV levels. dsRNA, double-stranded RNA; DWV, deformed wing virus; IRES, internal ribosome entry site; LP, leader protein; NS, not significant; Prot, viral protease; RdRpol, RNA-dependent RNA polymerase; RNAi, RNA interference; VP, structural viral protein; Vpg, genome-linked protein.

We sought to experimentally test whether natural variation between DWV genotypes coexisting in a single population was sufficient to significantly impact the effectiveness of antiviral RNAi and, indeed, could act as an RNAi evasion mechanism. It was shown previously that antiviral RNAi in honey bees could be induced by introduction of double-stranded RNA (dsRNA) molecules orally [30]. To do this, we produced dsRNA specific to two coexisting isolates, US DWV-304 and DWV-422. The targeted 283-nt region of DWV genomic RNA (positions 1,242–1,524 of the GenBank accession MG831200) had 5.3% divergent nucleotides between isolates DWV-304 and DWV-422 (S1 Text), which mostly resulted in one mismatch in any given 22-nt sliding window (Fig 7A). In two independent experiments (design summarized in Fig 7D), newly emerged adult honey bees from colonies with low *Varroa* infestation levels were fed with 1 μg of the dsRNA (ds304 or ds422) in 5 μL of 50% sucrose either with or without the virus extract containing 10^8^ genome equivalents of the clone-derived DWV-304 or DWV-422. In order to control for the impact of non-sequence-specific triggering of antiviral response by dsRNA, a phenomenon reported in honey bees [31,32], the two dsRNAs used were 94.7% identical at the nucleotide level and therefore likely to have similar nonspecific antiviral triggering capacity. The DWV-304- and DWV-422-derived dsRNAs therefore acted as controls against each other, making it possible to investigate the impact of the 5.3% nt variation between them on DWV infection dynamics. The bees were maintained for 7 days prior to sampling and quantification of DWV loads in individual insects. The identity of clone-derived DWV variants was confirmed by sequencing the RT-PCR fragments corresponding to the LP region.

The outcome of DWV-304 infection was significantly influenced by the specificity of the dsRNA. There were significantly higher levels of DWV in the DWV-304-infected bees, which received ds422 (Fig 7B and 7C, group DWV304/ds422), compared with the group that received ds304 (Fig 7B and 7C, group DWV-304/ds304), *P* < 0.05, ANOVA. This was observed in two independent experiments using honey bees from different colonies with different wild-type background DWV levels (Fig 7C and 7D, mock infected). Similar variation in “background” wild-type DWV loads was reported previously [33]. Virus levels across individual bees showed a bimodal distribution, which allowed us to categorize DWV loads as low or high, with a 10^9^-copy threshold, and carry out a 2 × 2 contingency table analysis. We found that, in the case of bees infected with DWV-304, a significantly reduced number of bees developed a high-level DWV infection in the group “DWV-304/ds304” (which received ds304 perfectly matching virus isolate) compared with the group “DWV-304/ds422” (which received ds422 mismatching the inoculated virus), *P* = 0.000185 (Fig 7D). Notably, the proportion of bees with high DWV levels in the group “DWV-304/ds304” were not significantly different from that in control group “Mock-infected/ds304” (Fig 7D). Similarly, ds422 suppressed development of high DWV levels more efficiently in the case of inoculation with DWV-422 compared with DWV-304, *P* = 0.0115 (Fig 7D, groups “DWV-304/ds422” and “DWV-422/ds422”), although it cannot be excluded that, in this case, differences of infectivity of these virus isolates might play a role in development of the observed pattern of infection. These results suggest that natural variation between DWV genotypes within a single population (e.g., DWV-304 and DWV-422) might significantly reduce the efficiency of RNAi targeting.

## Discussion

Analyses of DWV population genetics provides insights into factors that contribute to sharp increases in virulence. DWV populations with different degrees of virulence are available, and the emergence and spread of highly virulent DWV has been linked to the spread of the mite *V*. *destructor* [5,6]. *Varroa* appeared in the US in 1987 [34] and is currently detected across the US in 84.9% of migratory beekeeping operators and 97.0% of stationary apiaries [35], which reflects its global prevalence [5,11]. Although DWV is transmitted through vertical and oral routes [2], the mite provides an efficient route for horizontal transmission of DWV via direct injection into the honey bee’s tissues [10,13]. As an example of the impact of *Varroa* on DWV dynamics, low-level DWV infections were reported in the *Varroa*-free Hawaiian Islands, but introduction of *Varroa* mites led to a 100–1,000-fold increase in virus levels [10]. Importantly, the Hawaiian DWV diversity study [10] showed that only a few variants (if not a single variant) of DWV were found at high levels when transmitted by *Varroa* mites, reflecting a steep drop in virus genetic diversity. Similarly, in a separate study, it was shown that not all DWV variants present in the bees from *Varroa*-free Colonsay Island (Scotland, UK) were equally capable to establish high-level DWV infection when injected into the honey bee [13]. Moreover, generation of highly virulent *Varroa*-adapted DWV variants might involve large numbers of the mite-infested bees and considerable time to allow emergence of mutant variants of DWV that are virulent. This is supported by another study, which found that *Varroa* was present in honey bee colonies on the island of Fernando de Noronha for over 30 years, but DWV in the islands’ 20–40 honey bee colonies replicated only to low levels. It was suggested that, because of the small number of colonies, there was no chance to generate a virulent DWV variant. Importantly, the islands’ bees were susceptible to European virulent *Varroa*-selected DWV [36]. Through *Varroa*-mediated transmission, survival and propagation of DWV became less dependent on the survival of infected bees, thereby allowing for the propagation of viral variants with increased replication levels and virulence. It is unclear which genetic changes are associated with a *Varroa*-mediated transition, but selection of certain strains of DWV may be involved, such as recombinants between DWV-A and DWV-B [12–14,37]. Although both major DWV types, DWV-A and DWV-B, and their recombinants are vectored by *Varroa* mites [13,38], it is possible that DWV-B and the recombinants with the DWV-B-derived structural gene block are better adapted to mite transmission than DWV-A [12,13]. It was shown that DWV-A does not replicate in *Varroa* mites (it is vectored in a nonreplicative manner [38]); however, it cannot be excluded that replication of DWV-B does take place in the mite. DWV-B replication in the mites is suggested by its detection in the *Varroa* synganglion [39], as well as the observance of a higher proportion of DWV-B within a DWV population in pupae injected with a mite virus extract, compared with the virus population passaged via artificial injection in pupae [40]. Preferable mite vectoring of DWV-B compared with DWV-A could explain the rapid recent expansion of DWV-B in the US [19,41], from 3% of apiaries in 2010 to 66% of apiaries in 2016, whereas DWV-A was already ubiquitous in 2010 and was present in 93% of tested colonies in 2016 [19]. The spread of DWV-B in the US warrants further analysis of interactions between DWV-A and DWV-B, considering the possible emergence of highly virulent recombinants between these isolates, similar to those observed in the UK [12,13]. Indeed, such recombinant genomes, though at relatively low levels, were already detected in US bees [19]. Another marked change in DWV dynamics linked to *Varroa* was a dramatic reduction of genetic diversity in *Varroa*-transmitted DWV to nearly clonal virus populations in the UK in 2013 and the Hawaiian Islands in 2009, in stark contrast with high diversity of DWV in covertly infected, *Varroa*-free honey bees, which had low DWV levels [10,13]. These DWV diversity shifts were reported in two independent studies in different geographic locations, suggesting that low genetic diversity might be a universal feature of virulent *Varroa*-associated DWV. Surprisingly, in this study, we found that virulent DWV-A populations sampled in 2010 in Texas and Maryland, and in 2015 in Maryland, were highly divergent (Fig 1B). These DWV-A populations showed highly correlated distributions of the polymorphic sites (Fig 1B and S3 Table) and close phylogenetic relatedness between consensus sequences (S2 Fig). This might indicate that very similar DWV-A populations or even a single population currently circulate in the US *Varroa*-infested honey bee colonies or, at least, that such DWV population(s) are very common in the US. The DWV populations in individual bees sampled in 2015 in Maryland showed significantly higher diversity than the DWV in the UK 2013 study [13], which used the same high-throughput sequencing approach (Fig 2A). Diversity of DWV was equally high in individual US bees regardless of virus load (Fig 2, groups 3 and 4), whereas bees sampled in the UK in 2013 had a significantly lower virus diversity in the bees with high DWV loads (Fig 2A, groups 1 and 2).

Differences between UK and US DWV genetic diversity levels are dramatic when colony-level virus populations from pools of 50 bees are compared (Fig 2A and 2B). Genetic diversity of the UK and the US colony-level samples (50 bees) were not singificantly different from individual bees with high DWV levels in their respective locations (Fig 2A, UK 2013: *P* = 0.7408, US 2015: *P* = 0.326, ANOVA). This suggests that individual bees sampled in the UK were infected with the same or very similar DWV variants, whereas individuals in the US were infected with many divergent genetic variants of the virus. Indeed, similar distribution profiles of polymorphic positions were observed in the NGS libraries of individual bees and a colony-level population sampled in the US (Fig 1B and S3 Table), suggesting coexistence of the same multiple DWV genotypes even in individual US bees. Phylogenetic analysis of the full-length coding sequences of DWV genotypes from a single colony-level population sampled in 2015 showed a monophyletic origin of coexisting DWV isolates (S2 Fig, bootstrap value of 98%) and their genomic sections (Fig 3), indicating that this diversity was generated as a result of diversification of a single parental DWV genotype. Therefore, although samples from a period in time when low DWV diversity occurred in the mainland US have not been analyzed, there exists supporting evidence that a bottleneck did occur sometime prior to 2010, when high diversity was measured (Fig 1B, gray graph).

Another surprising outcome of our analysis of a DWV population using a full-length genome amplifications approach was the discovery of a high degree of divergence between some genotypes coexisting in the same virulent US DWV-A population. In some cases, this exceeded that reported for DWV isolates sampled in different locations several years apart. For example, coexisting US DWV isolates 702 and 306, with 1.66% nucleotide divergence, differ from each other more than recognized geographically distant isolates of DWV, such as the North American DWV-PA reported in 2006 [1] and DWV-*Vc*-Italy discovered in the *Vespa crabro* wasp in 2017 [42], with 1.52% of divergent nucleotides (S2 Fig). Such high heterogeneity in DWV populations highlights the importance of comprehensive characterization of RNA virus populations, and therefore, it should be considered when evolutionary histories of this virus are modeled based on phylogeography, especially because sampling is limited in many locations [11]. US DWV has significantly higher diversity when compared with other RNA viruses, including those infecting invertebrates. For example, the DWV cDNA clones from a typical individual colony, with a degree of diversity similar to that in individual honey bees (Fig 1B), had a 1.26% mean nucleotide diversity between the five infectious cloned isolates, whereas an assessment of cloned WNV fragments in the wild-collected mosquitoes and birds showed a mean interhost diversity of only 0.237% [43].

Further investigation of interactions between DWV genotypes within the virulent DWV-A population in a typical declining *Varroa*-infested US colony required isolation of individual components of the population. Because of the absence of DWV-free honey bee tissue culture for DWV [18], essential for isolation of the individual components of virus populations by “classical” plaque assays [44], we used a molecular approach to obtain DWV isolates. This involved the design of a series of infectious cDNA clones corresponding to individual RNA genomes forming a DWV population. Notably, all of the protein-coding sections of the DWV RNA in the cDNA clones were derived from the existing individual RNA molecules by using nearly full-genome amplification rather than being assembled, thereby constructing a “snapshot” of the quasispecies. In doing so, we effectively designed a reverse-genetics system that models an invertebrate RNA virus “mutant cloud” [45], which was the first of its kind.

One of the key drivers for diversification of DWV populations could be complementation between genotypes in mixed infections, as reported for other RNA viruses [20]. To test this hypothesis, we compared replication dynamics of individual isolates, combinations of the clones, and the parental wild-type DWV population by pupal injection test. Surprisingly, we established that at 3 dpi, virus levels were not significantly different in all cases, except the buffer (PBS) control (Fig 5B). In addition, the individual clone-derived isolates and wild-type DWV replicated to similarly high levels (Figs 4A and 5A) and showed the same replication dynamics, all reaching a plateau 24 hours after injection of virus particle extracts (Fig 5A). Taken together, this suggests that no complementation between genotypes of DWV population took place. At the same time, coinfection experiments with cloned isolates demonstrate their mutual compatibility and the lack of any strong competitive exclusion phenomena (S4 Fig). It should be noted that coexistence of divergent DWV isolates is not a rule. For example, in an experiment in which a DWV population sourced from a *Varroa*-free region containing DWV-VDV1 recombinants and DWV-A was injected into honey bee pupae, the recombinant isolates, usually associates with the *Varroa*-infested bees, overcompeted full-length DWV-A [13]. It is therefore very likely that US DWV isolates existing in the same population were adapted to each other in order to maintain high population diversity. We also established that recombination events between DWV isolates in a population were widespread, as evidenced by different topologies of the phylogenetic trees for genome sections of the full-length clones (Fig 3) as well as recombination events that were directly detected in virus progeny in the case of mixed infections of cloned isolates (Fig 5C and S4 Fig).

These results prompted questions about what drives diversification of DWV and the maintenance of high diversity in the absence of apparent complementation between isolates, as well as the contradiction between the high DWV diversity in the *Varroa*-infested US DWV population and the previously reported nearly clonal nature of the *Varroa*-associated DWV in the UK [13] and Hawaii [10]. Based on our findings and those of others, we proposed the following model of DWV diversity dynamics. First, the introduction of *Varroa* reduced DWV diversity through a selective sweep in favor of virus variants capable of being transmitted by *Varroa* mites from the diverse pre-*Varroa* population, as seen in bees from the Hawaiian island Oahu in 2009. Although it cannot be completely ruled out that *Varroa*-associated DWV variants may be introduced with the mite, this scenario is unlikely because partial viral RNA sequencing demonstrated that the *Varroa*-selected variant was present in pre-*Varroa* populations [10]. Notably, this model does not indicate that DWV variants selected as a result of DWV transmission have to replicate in *Varroa* mites; instead, it suggests that they have better stability in *Varroa* mites and/or better replication when injected directly into the honey bee hemolymph. Indeed, a recent study that used a clone-derived DWV-A variant, the same DWV type that was present in 2009 in Hawaii [10], showed that this variant does not replicate in mites at all; instead, *Varroa* vectoring occurs in a nonpropagative, and possibly nonpersistent, manner [38]. Therefore, it is very likely that *Varroa* transmission selected a virus strain from the pre-*Varroa* repertoire—i.e., *Varroa* introduction bottlenecked DWV population 1 year after *Varroa’s* arrival.

We further suggest that, following this selective sweep, a period of *Varroa*-adapted DWV strain diversification via point mutations and recombination occurs. This is supported by evidence of a monophyletic origin for the coexisting genotypes of the *Varroa*-associated DWV-A quasispecies in the mainland US about 30 years after *Varroa* arrival (Fig 3 and S2 Fig). We hypothesized that the main driver for such diversification might be the generation of a DWV population able to better circumvent genotype-specific antiviral defenses—in particular, RNAi responses. This model could explain the coexistence and maintenance of multiple genetic variants. Indeed, the distribution of divergent positions in the DWV genome suggests that the virus explores sequence space to maximize the proportion of the genome containing mismatches in potential 22-nt siRNA targets, enabling it to evade specific RNAi (Fig 6). We further experimentally demonstrated that the natural degree of variation observed between clones coexisting in a single population (e.g., clones 304 and 422) could result in such evasion of the strain-specific RNAi (Fig 7). In particular, RNAi induced by oral introduction of a dsRNA corresponding to a section of DWV genomes that originated from the same DWV populations showing only 5% nucleotide divergency (Fig 7A) operated significantly more efficiently in the case of complete match between the DWV genomic RNA and dsRNA (e.g., ds304/DWV-304 versus ds422/DWV-304, Fig 7B–7D). Results of the experiments in general support this hypothesis, although future studies will be required to investigate possible RNAi-driven DWV diversification.

The presence of alternate DWV variants potentially capable of evading specific RNAi, which may cover close to 80% of the genome in a colony-level DWV population in which alternative nucleotide levels are >1% (Fig 6), may enable the virus population to quickly respond to specific RNAi by increasing the proportions of genotypes with mismatches in the targeted region that are less-efficiently targeted (Fig 6). Notably, the genome distribution of DWV-specific siRNA in infected honey bees is not uniform—rather, it is dominated by tens of highly abundant siRNAs [13,24,37]; therefore, it is possible that not all parts of the viral genomic RNA are equally targeted. In addition, the high recombination rate observed within the DWV populations (Fig 5C and S4 Fig) could result in the assembly of novel genotypes composed of genome sections less efficiently targeted by a given set of siRNAs, thereby enabling RNAi evasion. Most notably, this scenario requires the ability of individual genotypes to be independent from each other, consistent with the equal replication rates of isolates (Figs 4A and 5A) and the lack of complementation as observed in the mixed infections (Fig 5B). In other words, maintaining high genetic diversity is advantageous for the virus because recombining and changing proportions of existing variants can occur much faster than the accumulation of mutations, thereby enabling an instantly changing composition of predominant genotypes in response to different pressures, including RNAi.

This model of DWV population dynamics, along with both previous and current findings, fully supports the view that mutability of RNA viruses is essential in maintaining virulent phenotypes [46]. A selective sweep of DWV following the introduction of the novel mite vector might favor the subsequent generation of novel *Varroa*-adapted variants with superior virulence and capacity to escape antiviral defenses (e.g., being targeting by specific RNAi established against predominant genotypes as shown for other invertebrate RNA viruses [27–29]) in a process similar to “punctuated immune escape” [47]. This process has previously been proposed for both vesicular stomatitis virus [48] and Influenza virus A [49, 50].

A rapid expansion of one of many low-frequency genetic variants of a virus, which occurs due to increased fitness, will results in the temporary reduction of overall virus population diversity [47]. Indeed, the very low diversity of DWV in the UK colony-level sample (Fig 2A and 2B, “Colony 50 bees, UK-2013”) suggests that population was sampled at the point of sharp reduction. Subsequently, DWV diversity reverts back to high levels through accumulation of mutations, ultimately establishing virus populations with multiple isolates that coexist even in individual insects (Fig 2A, group 4), with a high colony-level diversity (Fig 2A and 2B, “Colony 50 bees, USA-2015,” pointed with green arrow). Indeed, our initial analysis of DWV in 2015 samples from Oahu Island in Hawaii showed a high variability that matched the levels currently observed in the mainland US (S6 and S7 Figs). Thus, a long-term, semistable coexistence of virus isolates may arise spontaneously in virus populations as observed in bacteria [51]. It is notable that our phylogenetic trees along with our NGS evidence from 2010 suggest that the bottleneck in US mainland DWV diversity occurred before 2010 and that the introduction of *Varroa* to the US mainland occurred in the 1980s [11,34]. Although our study did not involve analysis of DWV from all US locations, it is very likely the DWV-A population we analyzed in depth was very common in the mainland US because it appeared in both 2010 and 2015 bees sourced 2,000 km apart in Texas and Maryland. Further analysis of additional samples collected shortly before and after the US mainland *Varroa* introduction, and/or samples from Hawaii prior to the *Varroa* introduction, would serve to confirm the role of *Varroa* in the selective sweep and significantly reinforce our model.

Our study of DWV has provided new insights into the dynamics of the divergent DWV population structure using a reverse-genetics system and high-throughput sequencing approaches. This system was particularly well suited to investigate interactions between viral genotypes within an invertebrate RNA virus quasispecies. The high genetic diversity of DWV and its potential to instantly respond to the pressures of sequence-specific RNA-mediated antiviral defense should be taken into account for the further development of antiviral interventions, including those based on RNAi [26] or superinfection exclusion (SIE) [52–55], which may be used to improve honey bee health and ensure sustainable food security [9]. Although antiviral RNAi can be induced in honey bees by oral introduction of DWV-specific dsRNA [30], the virus variants not perfectly targeted by the introduced dsRNA, which might already exist in the DWV population, might became abundant, thus reducing the antiviral effect. SIE on the other hand involves exclusion of a virus infection in the cells or organisms preinfected with a different strain of the same virus. The SIE mechanism may operate in several ways, including RNAi [56], and was observed in honey bees [57]. Given that DWV is a member of the family Iflaviridae [58], which infects species of multiple insect taxa [59], further analysis of the DWV gene functions and its interaction with the host, using our reverse-genetics system as developed in this study, could be applied to other viruses to further understand fundamental aspects of RNA virus infections and evolution in invertebrates and their wider ecological impact.

## Materials and methods

### Honey bees

*Varroa*-infested honey bee colonies maintained by the USDA-ARS Bee Research Laboratory (Beltsville, Maryland, USA), which had high *Varroa* infestation (15%–20% of pupae infested with mites), and bees showing wing deformities consistent with high DWV levels were used as the source of mite-exposed honey bee pupae for NGS analysis and isolation of DWV particles in October 2015. Unparasitized honey bee pupae from colonies with low *Varroa* infestation (with below 1% of mite-parasitized pupae) were used in the injection experiments. Newly emerged pupae from the Maryland colonies with low *Varroa* infestation were used in the oral infection experiments. A colony from Texas sourced in January 2017 was used in the in vitro RNA transcript and wild-type RNA injection experiment. A colony from Florida sourced in February 2017 was used in the experiment involving injection of virus extracts, both clone-derived and wild-type.

### Sequence analysis

The NGS libraries for this study were produced by Illumina HiSeq2500, with each containing 12–14 million paired-end reads (S1 Table; NCBI BioProject PRJNA431793, Sequence Read Archive study SRP135682). These libraries, along with the previously published UK NGS libraries [13] (S2 Table 2), were analyzed as described previously [19]. Briefly, Illumina reads were trimmed to remove adapters, contaminant sequences, and low-quality bases. Cleaned data from each sample library were individually aligned to reference sequences of three distinct strains of DWV-like viruses, DWV-A (GenBank accession GU109335), VDV1 (GenBank accession AY251269), and DWV-C [3], using Bowtie2. Nucleotide counts, coverage, and Shannon’s diversity index estimates for each nucleotide position were calculated from SAMtools mpileup output. Although Illumina HiSeq technology has a very low per-nucleotide substitution error rate (0.08% and 0.12% for the first and second reads, correspondingly [60]), to reduce sequencing chemistry errors, the Shannon’s diversity index estimate was further corrected for potential errors using the approach described previously [61]. In brief, the error model was calibrated using data from an Illumina library from a bee injected with a cloned virus of known sequence. Data from positions 5,230 to 6,600 of the DWV genomic RNA, representing a low-diversity region of the virus, were used to calculate an alphahat of 9.88 × 10^−5^. To quantitatively assess the degree of similarity between different DWV genetic diversity profiles, we calculated the Pearson’s correlation between the Shannon’s diversity index profiles averaged for 100-nt windows. For detection of recombination breakpoints in the honey bee pupae injected with the mixtures of the clone-derived DWV genotypes, cleaned data from each sample NGS library were individually aligned to the VDV1 and DWV references using SpeedSeq (version 0.1.2). Structural variants were detected using the lumpy smooth script from the LUMPY package (version 0.2.13), and additional genotype and coverage metrics for each variant were calculated using SVTyper, a script within the SpeedSeq package. Breakpoint positions representing a recombination between two different viral isolate sequences were considered for further analysis if they had evidence from more than 10 supporting events (split or discordant reads) and exceeded 0.02% of NGS coverage (the level was chosen to ensure that the reads corresponded to viable—i.e., replicating—recombinant genomes, rather than aberrant variants). Phylogenetic analysis was carried out by aligning the viral nucleotide sequences using CLUSTAL and producing maximum likelihood and neighbor-joining trees, which were bootstrapped using 1,000 replicates with the RAcML and PHYLIP packages, respectively. dS and dN variations in viral genomes were identified using all known full-length North and South American DWV-A sequences (S2 Fig) using tools provided by the Los Alamos National Laboratory’s HIV Databases (https://www.hiv.lanl.gov [62]); putative positive diversifying selection was assumed when dS/dN < 1 (S4 Table).

Analysis of DWV diversity in Hawaiian (Oahu Island) apiary-level samples collected in 2015 as a part of the USDA-APHIS honey bee survey [35] included qRT-PCR amplification of the DWV cDNA fragments corresponding to the LP and the RdRp regions, using the oligonucleotide primers “DWV-LP-For” and “DWV-LP-Rev”, “DWV-POL-For” and “DWV-POL-Rev” (S5 Table), respectively, and direct sequencing of the RT-PCR products to identify polymorphic sites in the electropherograms. The RT-PCR fragments corresponding to the LP region were also cloned into a pGem-T easy vector (Promega) and Sanger sequenced.

### DWV cDNA clones and clone-derived viruses

Viral particles were purified by caesium chloride gradient centrifugation from a pool of 50 pupae out of a single *Varroa*-infested colony at the Bee Research Lab in Beltsville, Maryland, USA, in October 2015 as described previously [12]. Viral RNA was extracted from the virus preparation using the RNeasy kit (Qiagen). The first cDNA strand corresponding to the entire 10-kb-long genomic RNA was produced using Superscript III Reverse Transcriptase (Invitrogen) and the oligonucleotide primer “DWV-PmeI-A27Rev” complementary to the 3′ terminus of the DWV genomic RNA, which was preceded by the 27-nt oligo dT and the *Pme*I restriction site (S5 Table). The cDNA was used as a template to amplify a nearly full-length, 9.7-kb DWV RT-PCR fragment containing the 686-nt section of 5′ untranslated region adjacent to the viral ORF, the full 8,682-nt protein-coding sequence, and complete 341-nt 3′ untranslated region using the oligonucleotide primers “DWV-PmeI-A27Rev” and “DWV-USA-PacI462F,” identical to positions 472–502, preceded by the *Pac*I restriction site (S5 Table), and the proof-reading thermostable DNA polymerase Phusion (New England Biolabs). The library was cloned into the pCR4Blunt-TOPO vector (Invitrogen) according to the manufacturer’s instructions. Ten clones containing 9.7-kb viral cDNA inserts were Sanger sequenced (GenBank accession numbers MG831200–MG831204 and MH069503–MH069507). A set of five divergent DWV cDNA constructs (GenBank accession numbers MG831200–MG831204) was selected to construct a set of full-length infectious DWV cDNA clones capturing a significant proportion of colony-level diversity. This was accomplished by inserting at the position corresponding to the 5′ part of the DWV cDNA the synthetic fragment “T7-Ribo-5′-DWV” (S5 Table) containing the T7 RNA polymerase promoter sequence, the 5′ ribozyme sequence [63], and the 462-nt-long 5′ terminal part of the DWV genome, using the *Not*I and *Pac*I restriction sites to create a series of five plasmids with different full-length cDNA inserts. The 462-nt-long 5′ terminal sequence was designed according to the DWV USA NGS data obtained in this study and included an extended 5′ terminal sequence (as previously [64] and in the GenBank accession number KT215904). To produce control constructs p304Δ and p306Δ containing noninfectious DWV cDNA, the regions 7,128–7,998 and 7,128–8,347 coding for the viral protease region were deleted from clones p304 and p306 (GenBank accession numbers MG831200 and MG831201), respectively, creating a frameshift in the viral ORF that abolished translation of the viral RdRpol. Plasmid constructs were linearized by using the *Pme*I restriction site located at the 3′ end of DWV cDNA, downstream of the A27 sequence, to produce the templates for a full-length 10.2-kb transcript. The in vitro transcripts were produced using HiScribe T7 High Yield RNA Synthesis Kit (New England Biolabs) according to the manufacturer’s instructions. After 3 hours of incubation at +37 °C, the DNA templates were digested using TURBO DNase (Life Technologies). The RNA transcripts were purified using the RNeasy mini kit (Qiagen), eluted with RNAse-free sterile water, and stored at −80 °C prior to use.

RNA transcripts were injected into honey bee pupae without *Varroa* mite feeding exposure. These pupae were extracted from brood frames with low *Varroa* infestation (approximately one mite-infested cell per 500) from a Texas apiary in December 2016. The pupae at pink eye stage were injected intra-abdominally into the hemolymph with 5 μg (13.2 × 10^12^ copies) of the in vitro transcripts or wild-type DWV RNA in 10 μL of PBS or 10 μL of PBS control using syringes with a 0.3-mm needle G31 (BD Micro-Fine), essentially as described previously [13], and were incubated at +33 °C, relative humidity 80%, for 3 days to allow replication of the virus prior to RNA extraction and sequencing. It was necessary to inject high copy numbers of naked viral RNA to reliably initiate clone-derived infection because, as expected, infectivity of naked RNA was very low compared with an encapsidated RNA. Notably, the injected RNA, either in vitro transcript or extracted from virus particles, was almost entirely degraded in the hemolymph after 3 days; qRT-PCR quantification of DWV in the pupae injected with 9.3 × 10^12^ copies of the control nonreplicating DWV in vitro transcripts p304Δ and p306Δ had between 1.3 × 10^5^ and 1.7 × 10^7^ genome copies detected (Fig 4A). For comprehensive characterization of the transcript-derived progeny, NGS libraries were generated from individual pupae each containing 12–17 million paired-end 150-nt reads, with the share of DWV reads ranging between 43% and 63% (S1 Table 1).

For preparation of the clonal DWV extracts containing infectious DWV virus particles, individual transcript-injected pupae were homogenized with 2 mL of PBS at 3 dpi. For each individual pupal extract, 1 mL was used for preparation of the DWV inocula, which included clarification by centrifugation at 3,000*g* for 5 minutes and filtration through a 0.22 μm nylon filter (Millipore). The DWV concentration in the extracts was quantified by qRT-PCR, and the extracts were stored at −80 °C prior to use. To confirm the identity of the clonal DWV in the extracts with their respective parental DWV cDNA clones, the DWV RNA region, positions 30–1,266 nt, was amplified by RT-PCR, sequenced, and also digested with *Pac*I restriction enzyme.

Pink eye–stage honey bee pupae, with no exposure to *Varroa* mite feeding, were extracted from a Florida brood frame with low *Varroa* infestation (1 infested cell per 500) in January 2017. Pupae were intra-abdominally injected into the hemolymph to introduce either the filtered DWV extracts (10^7^ genome copies in 10 μL of PBS), which included the clone-derived recovered DWV variants that originated from transcript-injected pupae, the wild-type DWV, or buffer control (PBS). We sampled time series from “time 0” (immediately after injection) through to 3 dpi, and the DWV RNA copy numbers were quantified in individual bees by qRT-PCR as described previously [38].

### Assessment of the effect of strain-specific dsRNA on DWV replication

dsRNAs specific to the DWV clones were produced in vitro with T7 RNA polymerase (HiScribe T7 RNA polymerase, New England Biolabs) using as the templates PCR fragments corresponding to the positions 1,242–1,524 of the clones DWV-304 and DWV-422 (GenBank accession numbers MG831200 and MG831202, respectively) with T7 promoter sequences at both the 5′ and 3′ termini. These PCR fragments were amplified using the US DWV cDNA clones p304 and p422 as the templates and the primers shown in S5 Table. Oral infection included feeding individual newly emerged bees with 5 μL of 50% sucrose containing 1 μg of purified dsRNA (ds304 or ds422) and 10^8^ clone-derived virus particles, DWV-304 or DWV-422, or no virus in the case of control groups (Fig 7D). The honey bees were sourced from Maryland colonies with low *Varroa* infestation levels (less than 1% of pupae being infested with the *Varroa* mites) and were starved for an additional 3 hours before feeding. The bees were kept for 7 days essentially as described in [65] in cages at +33 °C, 75% relative humidity, with *ad libitum* sources of water and 1:1 sucrose/water syrup (w/v), prior to RNA extraction.

### Data deposition

Raw sequence data and consensus DWV sequences have been deposited at DDBJ/EMBL/GenBank within BioProject PRJNA431793 under the Sequence Read Archive (SRA) study SRP135682 (accessions SRR6833910–SRR6833954) and Transcriptome Shotgun Assembly (TSA) study GGSE00000000 (accessions GGSE01000001–GGSE01000045), respectively. Cloned viral cDNA sequences are available in GenBank with accession numbers MG831200–MG831204, MH069503–MH069507, and MH594118–MH594121.

## Supporting information

S1 Fig Analysis of the US and the UK NGS libraries presented in S2 Table. NGS DWV-A read coverage (left panels) and Shannon’s diversity profiles (right panels) for the nonstructural gene region and 3′ UTR regions analyzed in Fig 2 are shown. X-Axes, positions in the DWV-A reference. The libraries are grouped according to Fig 2. DWV, deformed wing virus; NGS, next-generation sequencing.
(PDF)

S2 Fig Phylogeny of the complete coding sequence of DWV genomic RNAs. A maximum likelihood phylogenetic tree was generated for a 9.7-kb DWV genome section (position 472 in the 3′ IRES to the 3′ poly(A) sequence, position 10,162), which includes the entire protein-coding sequence. Bootstrap values for 1,000 replicates are shown for the groups with more than 50% bootstrap support. Red nodes connect the cloned sequences from a Maryland *Varroa*-infested colony sampled in 2015 (“Clone-”). Stars indicate the DWV cDNA clones designed in this experiment and tested for infectivity. Suffix “-Cons” indicates consensus NGS sequences. Green nodes and labels indicate NGS consensus sequences for DWV from individual honey bee pupae. Scale shows genetic distance (%). Isolates are labeled with their clone identifier or country of origin as well as their NCBI accession. DWV, deformed wing virus; IRES, internal ribosome entry site; NCBI, The National Center for Biotechnology Information; NGS, next-generation sequencing.
(TIFF)

S3 Fig Transmission electron microscopy of wild-type and cDNA clone-derived DWV-A virus particles. (A, B) Purified wild-type DWV-A virus particles isolated from *Varroa*-infested honey bee pupae. (C-E) Clone-derived DWV-A virus particles in filtered tissue extracts from honey bee pupa injected with in vitro transcript from full-length DWV-A cDNA clone 304. The virus particles on carbon-coated grids were negatively stained with sodium 1% phosphotungstic acid and observed at 80 KV with a Hitachi HT-7700 transmission electron microscope. The arrows indicate some filled DWV-A particles in virus preparations (A, B) and all DWV-A particles in the filtered extracts (C-E). Bar, 100 nm. DWV, deformed wing virus.
(TIFF)

S4 Fig Analysis of the within-population competitiveness and recombination of DWV genotypes. Individual honey bee pupae were injected with two separate clone-derived DWV isolates, 5 × 10^6^ copies of each, and analyzed by NGS at 3 dpi. Overall ratios between the clone-derived DWV isolates in the progeny, calculated as an average of the expected polymorphic nucleotide proportions ± SD, are shown for each combination. The top panels show recombination breakpoints, identified using the LUMPY package, and their proportions in relation to the NGS read coverage. Only the recombination sites with more than 10 supporting events exceeding 0.02% of the NGS coverage are shown; black and red diamonds show the recombination sites (black for the predominant isolate at 5′, red for the predominant isolate at 3′). In the lower panels, the orange bars show ratios between the expected polymorphic nucleotides for each pairwise combination along with trend lines. The black bars show the ratios of the second most abundant nucleotide to the total coverage at the positions, which were not polymorphic for a given genotype pair, indicative of the background wild-type DWV infection. For each graph, x-axes show nucleotide position in the DWV genome and y-axes show recombinant read proportions (top panes) or polymorphic nucleotide ratios (bottom panes). dpi, days postinoculation; DWV, deformed wing virus; NGS, next-generation sequencing.
(TIFF)

S5 Fig Diversifying selection of DWV proteins. Alignment shows divergent positions in the full-length North and South American DWV sequences and in the infectious US DWV cDNA clones. Codon numbers are indicated below. Positions marked with “+” correspond to putative codons under positive diversifying selection in DWV strain from Pennsylvania (AY292384), which is used as the reference genotype. DWV, deformed wing virus.
(TIFF)

S6 Fig Polymorphisms in *Varroa*-associated DWV from 2015 Hawaii and Maryland honey bee samples. Electropherograms of the direct sequencing of RT-PCR fragments corresponding to sections of DWV RNA: (A-C) LP region (positions 1,045–1,158) and (D-F) RdRpol fragment (positions 8,645–8,744, sequenced in the Hawaiian DWV diversity study to demonstrate DWV clonality in Oahu Island DWV in 2009 [9]). The fragments were amplified using RNA extracted from (A, D) pooled honey bees collected from a *Varroa*-infested apiary on Oahu Island, Hawaii, in 2015; (B, E) an individual honey bee pupa injected with clone-derived DWV-304; (C, F) an individual honey bee pupa infected with Maryland wild-type DWV (2015 sample source). Divergent positions are indicated with arrows. DWV, deformed wing virus; LP, leader protein; RdRpol, RNA-dependent RNA polymerase; RT-PCR, reverse-transcription PCR.
(TIFF)

S7 Fig Phylogenetic analysis of the LP-coding sequences of DWV RNA. Maximum likelihood phylogenetic tree corresponds to the section of the DWV genome coding for the LP and adjacent parts of 5′ IRES and the structural gene block (positions 922–2,048). Blue nodes connect the cloned sequences from a Hawaiian apiary (Oahu Island) sampled in 2015; red nodes connect the cloned sequences and the full-length DWV genomes from a Maryland *Varroa*-infested colony sampled in 2015. Bootstrap values for 1,000 replicates are shown for the groups with more than 50% bootstrap support. Scale bar shows genetic distance (%). Isolates are labeled with their clone identifier or country of origin as well as their NCBI accession. DWV, deformed wing virus; IRES, internal ribosome entry site; LP, leader protein; NCBI, The National Center for Biotechnology Information.
(TIFF)

S1 Text dsRNAs specific to the cloned DWV genotypes. Nucleotide alignment of the 283-nt section of DWV isolates DWV-304 and DWV-422 (GenBank accession numbers MG831200 and MG83120), positions 1,242–1,524 in the DWV genomic RNA, which were used to generate dsRNA. dsRNA, double-stranded RNA; DWV, deformed wing virus.
(PDF)

S1 Table NGS libraries. NCBI Sequence Read Archive accession SRP135682, https://www.ncbi.nlm.nih.gov/sra/SRP135682. NCBI, The National Center for Biotechnology Information; NGS, next-generation sequencing.
(XLSX)

S2 Table NGS summary and average Shannon's diversity of the NS and RdRp regions of the US and UK DWV populations. DWV, deformed wing virus; NGS, next-generation sequencing; NS, nonstructural; RdRp, RNA-dependent RNA polymerase.
(XLSX)

S3 Table Comparison of the distribution of divergent nucleotides in DWV populations. Pearson’s correlation for Shannon's diversity profiles for a 100-nt window. The degree of shade indicates the correlation coefficients: no shade, below 0.5; light shade, above 0.5–0.7; medium shade, from 0.7 to 0.9; and dark shade, above 0.9. DWV, deformed wing virus.
(XLSX)

S4 Table Synonymous and nonsynonymous substitutions in the infectious DWV clones. DWV, deformed wing virus.
(XLSX)

S5 Table Primers and the synthetic gene used in this study. (XLSX)

S1 Data Numerical values underlying the summary graphs in the figures. Separate data sheets of this Excel document, which are accordingly named, contain data for Figs 2A, 2B, 4A, 5A, 5B, 5C, 7B and 7C.
(XLSX)

## Abbreviations

dN: nonsynonymous
dpi: days postinoculation
dS: synonymous
dsRNA: double-stranded RNA
DWV: deformed wing virus
IRES: internal ribosome entry site
LP: leader protein
NGS: next-generation sequencing
qRT-PCR: quantitative RT-PCR
RdRpol: RNA-dependent RNA polymerase
RNAi: RNA interference
RT-PCR: reverse-transcription PCR
SIE: superinfection exclusion
siRNA: small interfering RNA
VDV1: *Varroa destructor* virus 1
VP: structural viral protein
Vpg: genome-linked protein
WNV: West Nile virus
