# Supplementary material for: Dynamic evolution in the key honey bee pathogen deformed wing virus: Novel insights into virulence and competition using reverse genetics
Source: PLoS Biol. 2019 Oct 10;17(10):e3000502. doi: 10.1371/journal.pbio.3000502 (PMC6805011; doi:10.1371/journal.pbio.3000502)
Supplement: S1 Fig — NGS DWV-A read coverage (left panels) and Shannon’s diversity profiles (right panels) for the nonstructural gene region and 3′ UTR regions analyzed in Fig 2 are shown. X-Axes, positions in the DWV-A reference. The libraries are grouped according to Fig 2. DWV, deformed wing virus; NGS, next-generation sequencing. (PDF) [file pbio.3000502.s001.pdf]

Colony USA 2015.

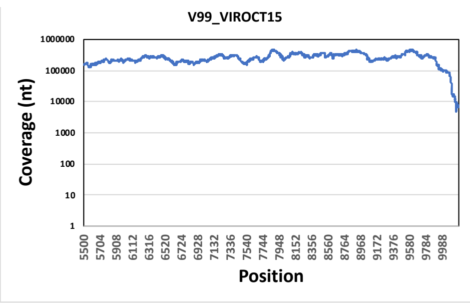

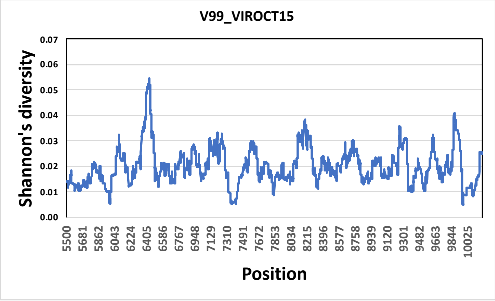

Colony UK 2013.

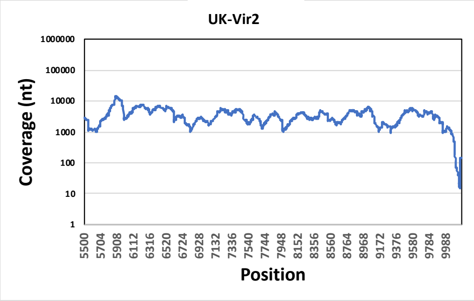

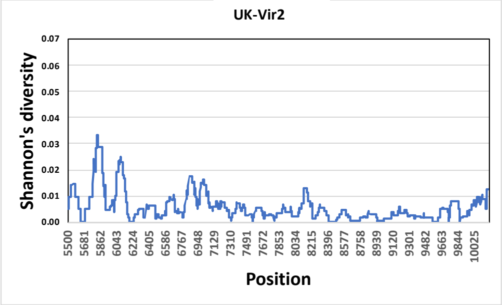

Group 1. UK 2013 - Wild-type - Low DWV

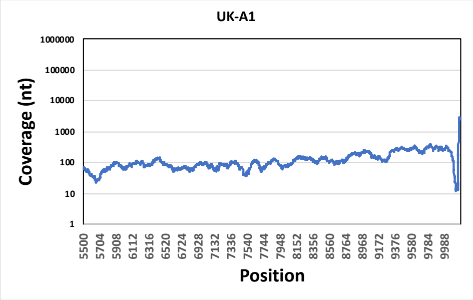

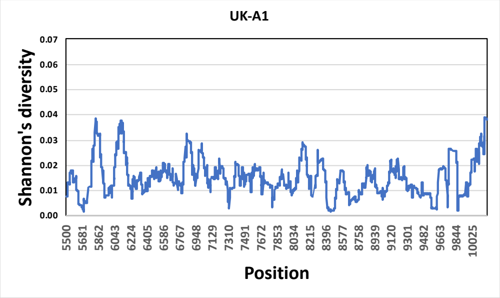

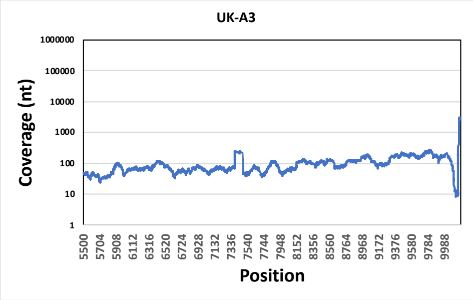

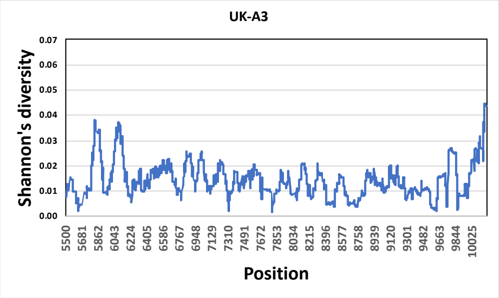

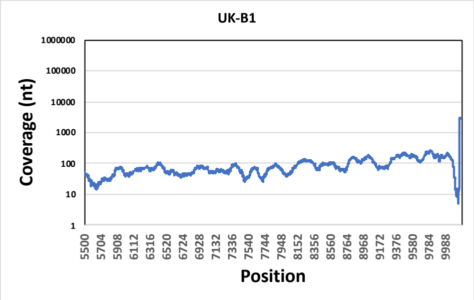

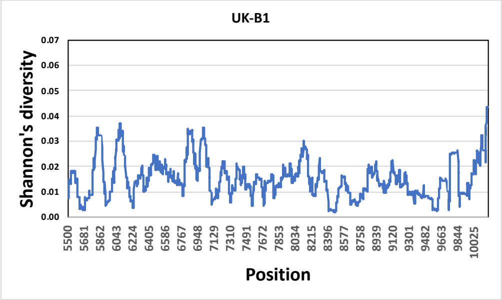

(continued on next page)

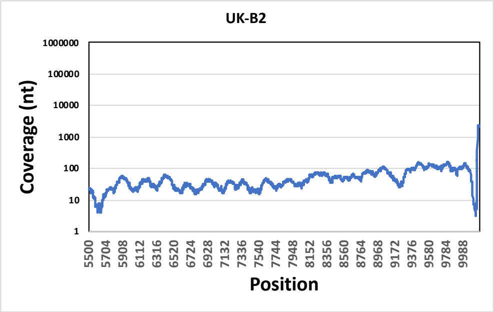

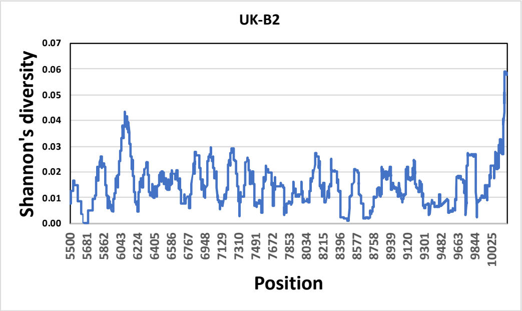

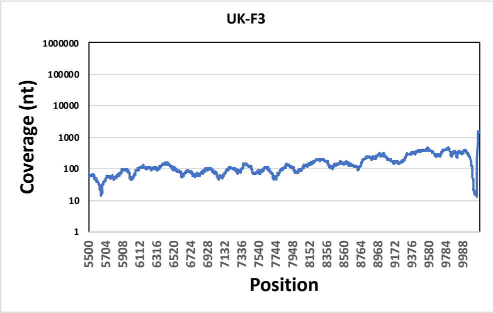

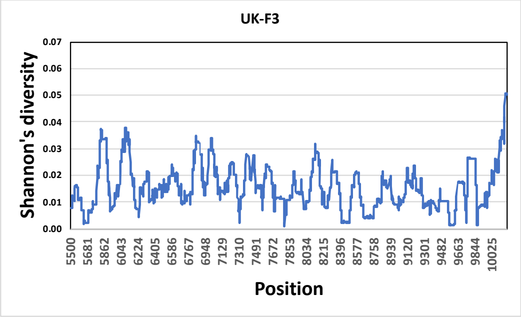

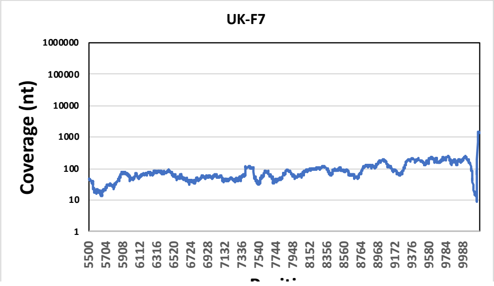

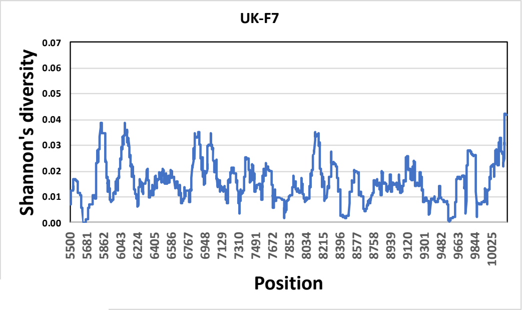

Group 2. UK 2013 - Wild-type - High DWV

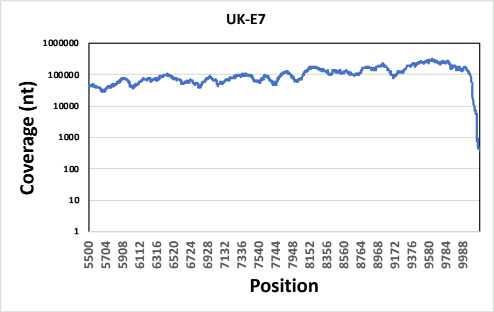

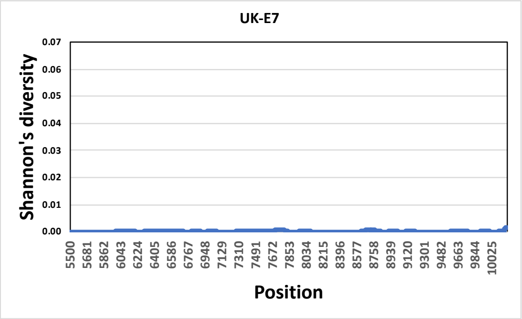

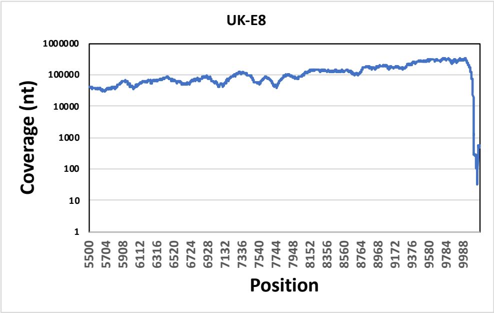

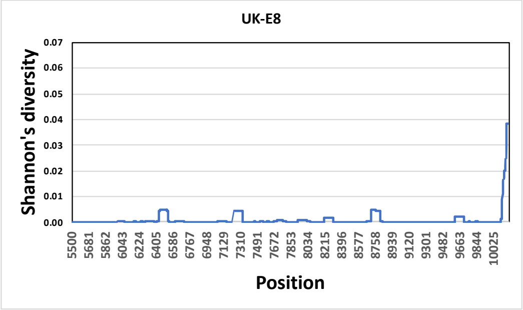

((continued on next page)

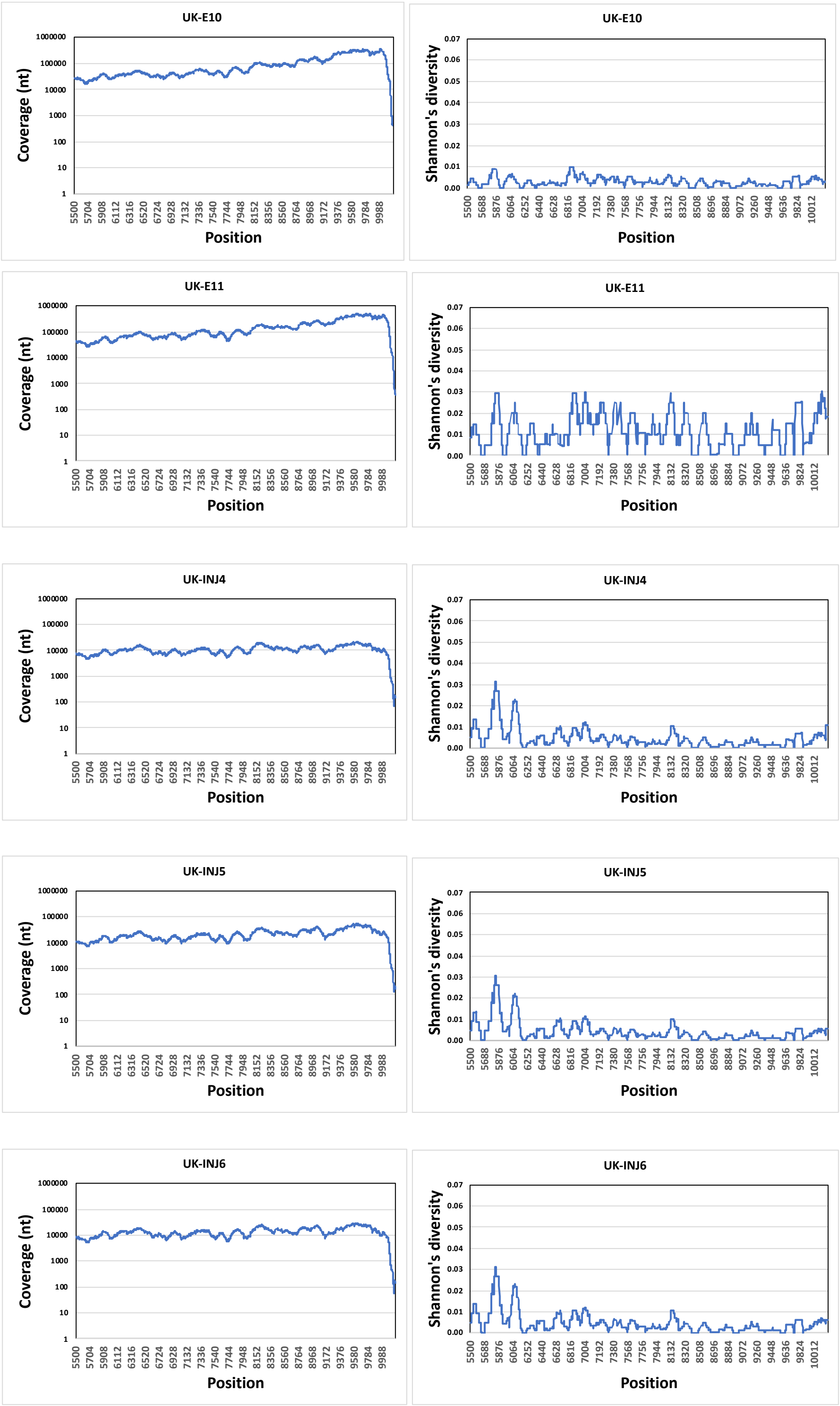

(continued on next page)

Group 3. USA 2015-2017 - Wild-type – Low DWV

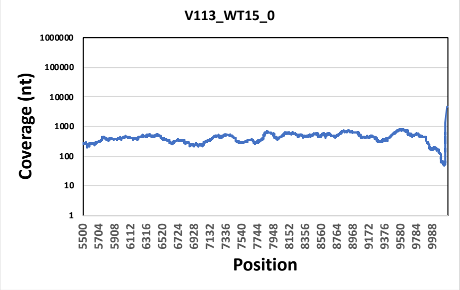

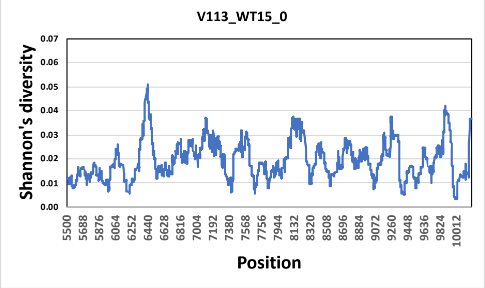

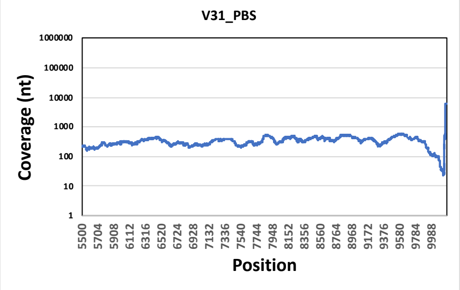

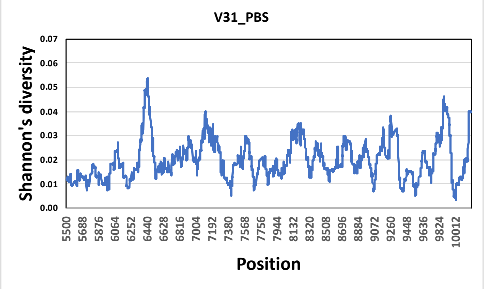

Group 4. USA 2015-2017 - Wild-type – High DWV

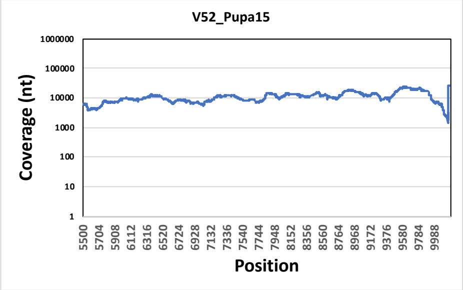

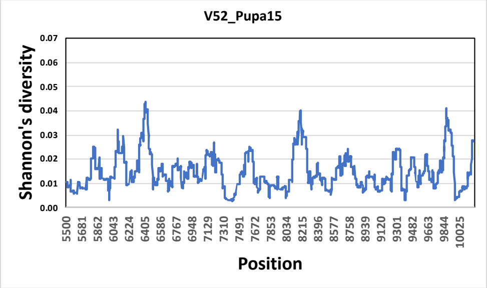

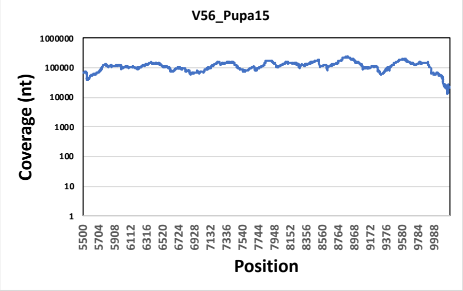

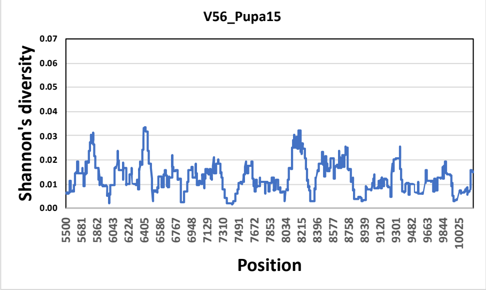

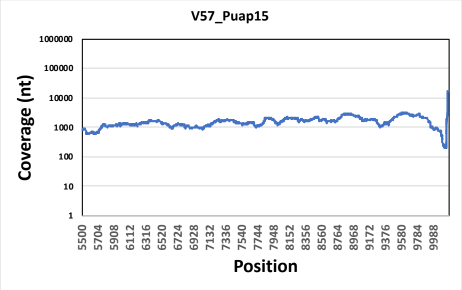

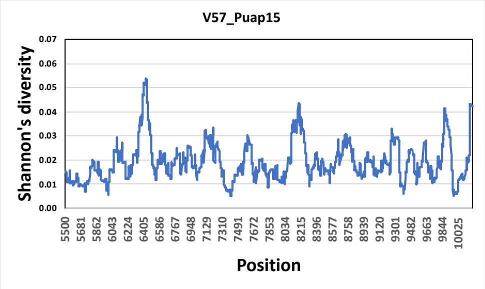

(continued on next page)

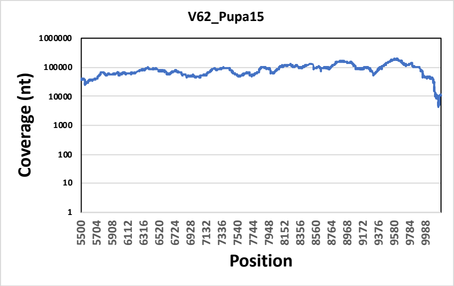

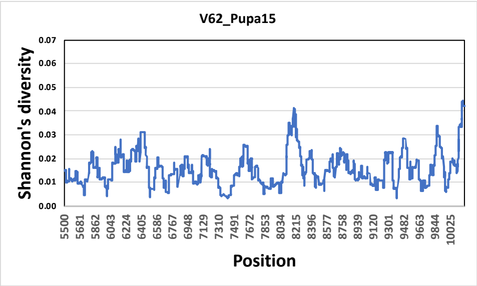

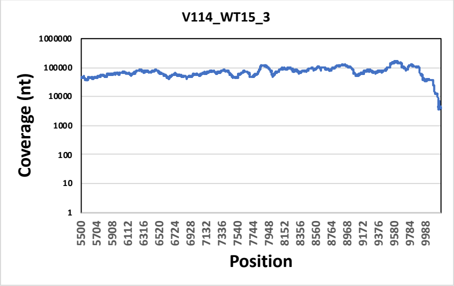

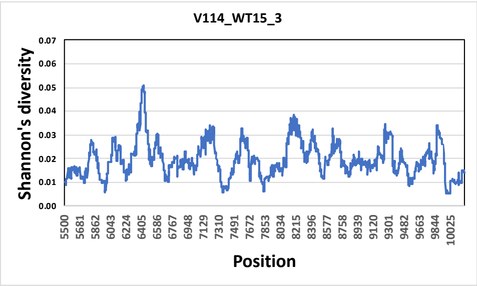

Group 5. USA 2015 - cDNA clone-derived - High DWV

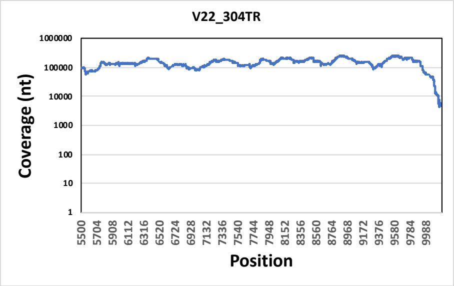

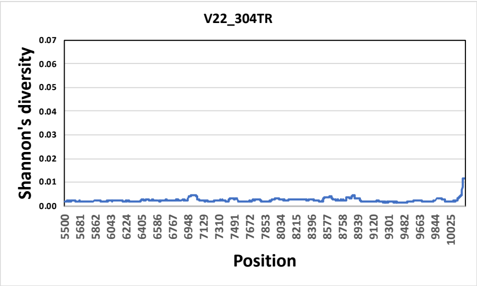

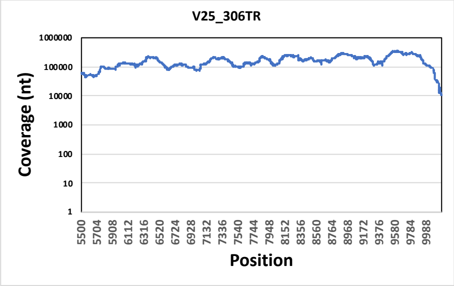

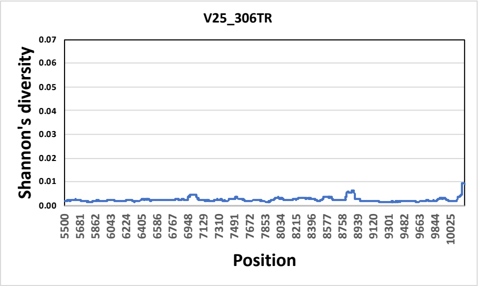

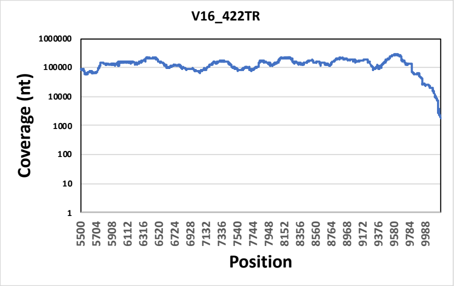

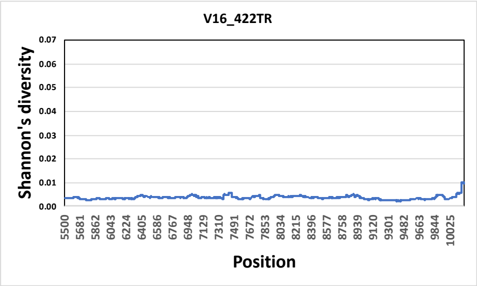

(continued on next page)

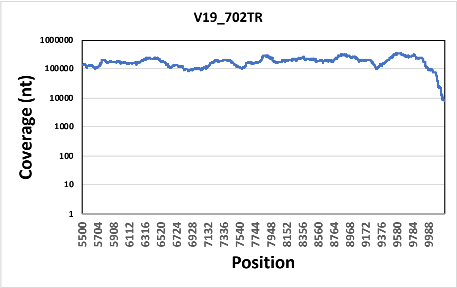
V19_702TR
Coverage (nt)
1000000
100000
10000
1000
100
10
1
5500 5704 5908 6112 6316 6520 6724 6928 7132 7336 7540 7744 7948 8152 8356 8560 8764 8968 9172 9376 9580 9784 9988
Position

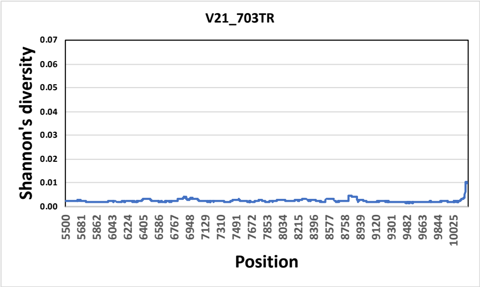
V21_703TR
Shannon's diversity
0.07
0.06
0.05
0.04
0.03
0.02
0.01
0.00
5500 5681 5862 6043 6224 6405 6586 6767 6948 7129 7310 7491 7672 7853 8034 8215 8396 8577 8758 8939 9120 9301 9482 9663 9844 10025
Position

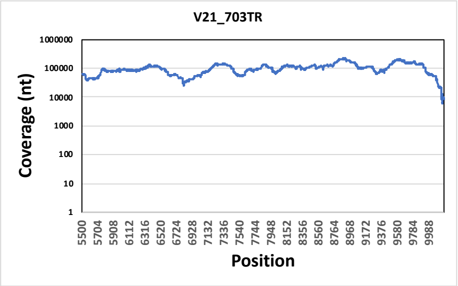
V21_703TR
Coverage (nt)
1000000
100000
10000
1000
100
10
1
5500 5704 5908 6112 6316 6520 6724 6928 7132 7336 7540 7744 7948 8152 8356 8560 8764 8968 9172 9376 9580 9784 9988
Position

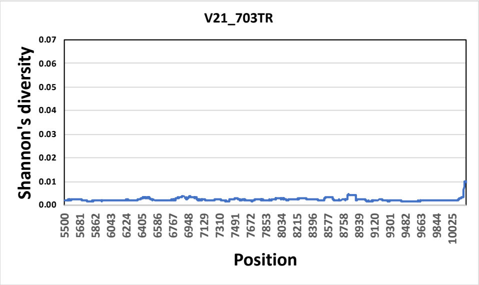
V21_703TR
Shannon's diversity
0.07
0.06
0.05
0.04
0.03
0.02
0.01
0.00
5500 5681 5862 6043 6224 6405 6586 6767 6948 7129 7310 7491 7672 7853 8034 8215 8396 8577 8758 8939 9120 9301 9482 9663 9844 10025
Position
