# Supplementary figures and images for: Dynamic evolution in the key honey bee pathogen deformed wing virus: Novel insights into virulence and competition using reverse genetics
Source: PLoS Biol. 2019 Oct 10;17(10):e3000502. doi: 10.1371/journal.pbio.3000502 (PMC6805011; doi:10.1371/journal.pbio.3000502)

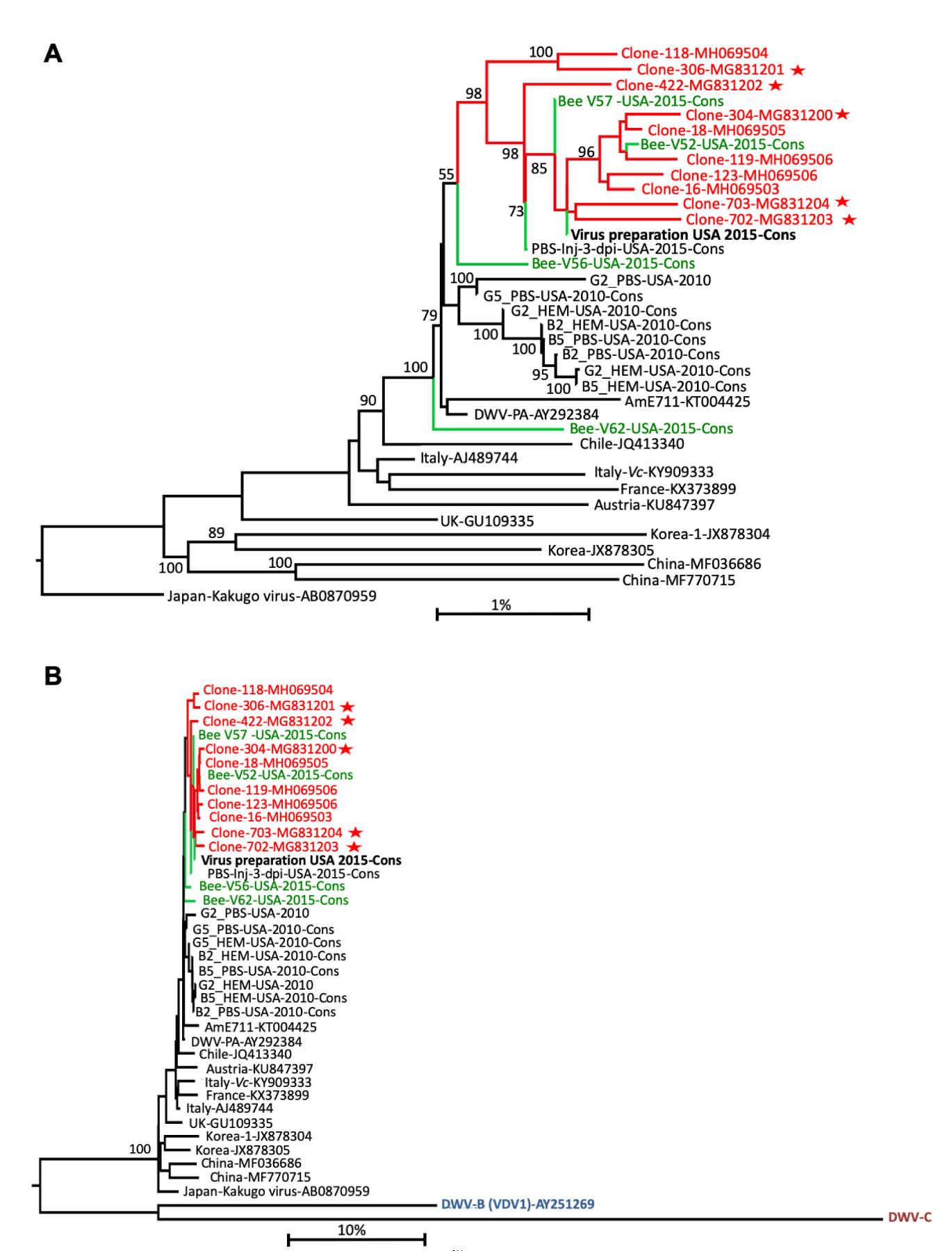

Supplement: S2 Fig — A maximum likelihood phylogenetic tree was generated for a 9.7-kb DWV genome section (position 472 in the 3′ IRES to the 3′ poly(A) sequence, position 10,162), which includes the entire protein-coding sequence. Bootstrap values for 1,000 replicates are shown for the groups with more than 50% bootstrap support. Red nodes connect the cloned sequences from a Maryland Varroa-infested colony sampled in 2015 (“Clone-”). Stars indicate the DWV cDNA clones designed in this experiment and tested for infectivity. Suffix “-Cons” indicates consensus NGS sequences. Green nodes and labels indicate NGS consensus sequences for DWV from individual honey bee pupae. Scale shows genetic distance (%). Isolates are labeled with their clone identifier or country of origin as well as their NCBI accession. DWV, deformed wing virus; IRES, internal ribosome entry site; NCBI, The National Center for Biotechnology Information; NGS, next-generation sequencing. (TIFF) [file pbio.3000502.s002.tiff]

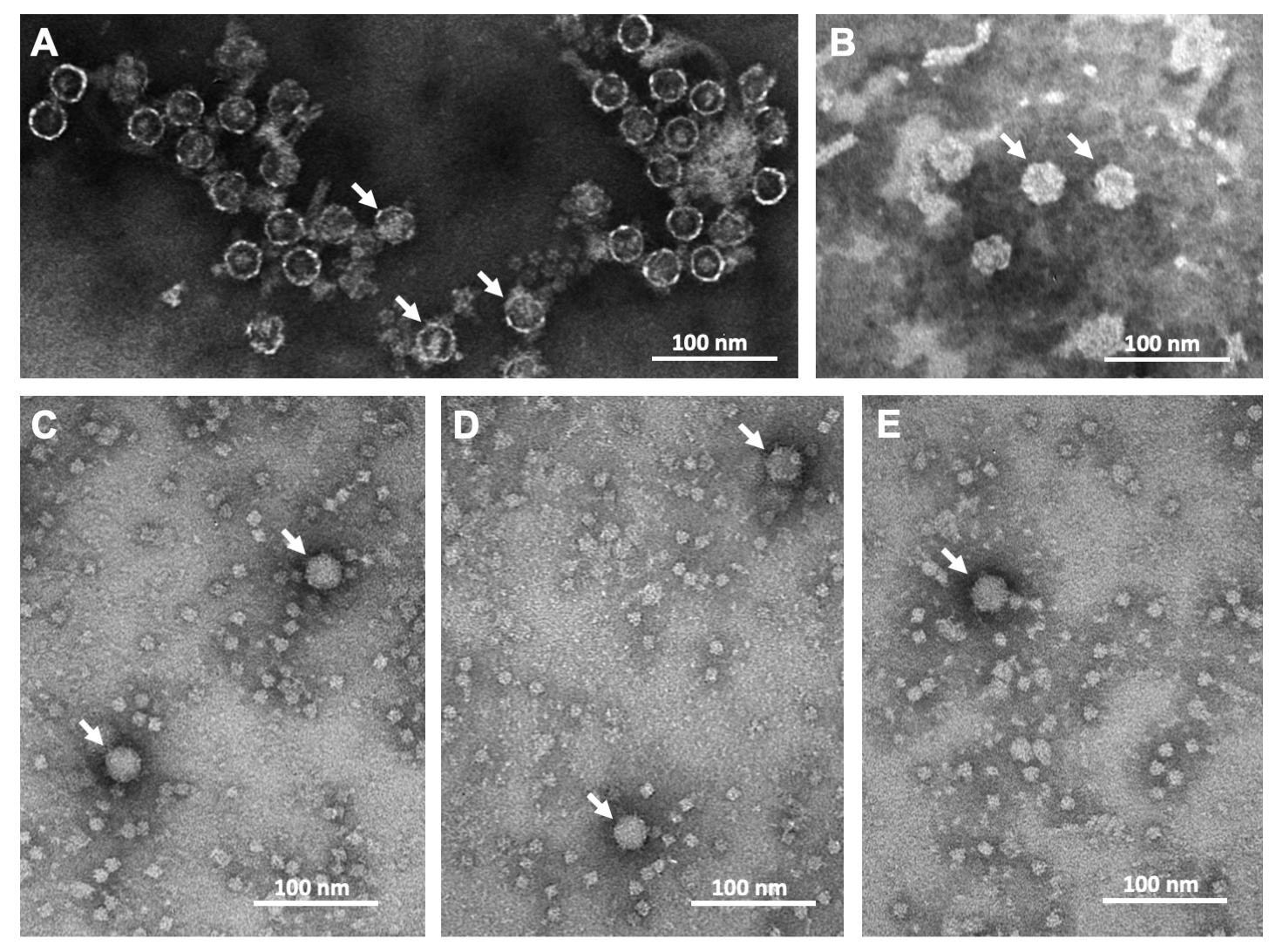

Supplement: S3 Fig — (A, B) Purified wild-type DWV-A virus particles isolated from Varroa-infested honey bee pupae. (C-E) Clone-derived DWV-A virus particles in filtered tissue extracts from honey bee pupa injected with in vitro transcript from full-length DWV-A cDNA clone 304. The virus particles on carbon-coated grids were negatively stained with sodium 1% phosphotungstic acid and observed at 80 KV with a Hitachi HT-7700 transmission electron microscope. The arrows indicate some filled DWV-A particles in virus preparations (A, B) and all DWV-A particles in the filtered extracts (C-E). Bar, 100 nm. DWV, deformed wing virus. (TIFF) [file pbio.3000502.s003.tiff]

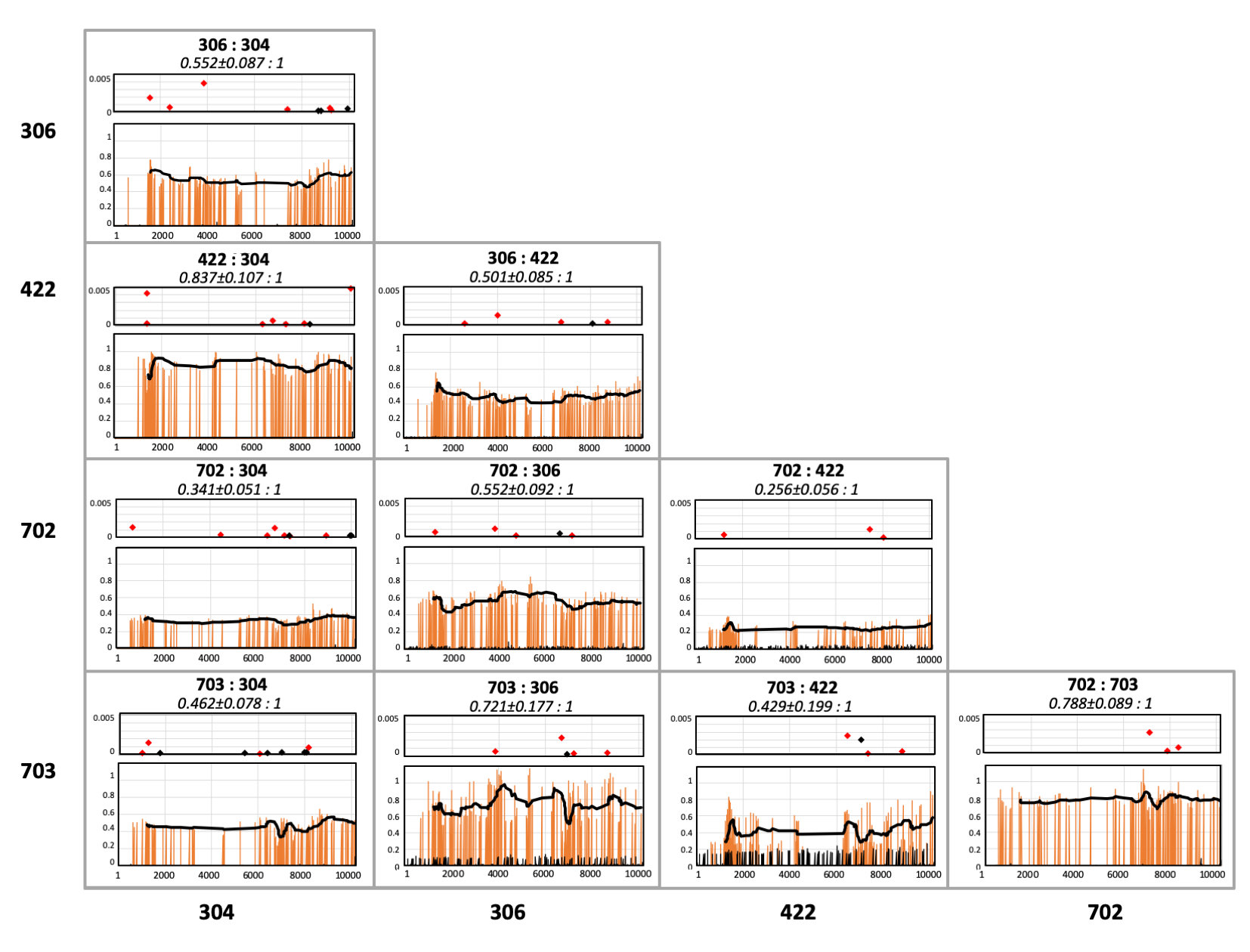

Supplement: S4 Fig — Individual honey bee pupae were injected with two separate clone-derived DWV isolates, 5 × 10^6^ copies of each, and analyzed by NGS at 3 dpi. Overall ratios between the clone-derived DWV isolates in the progeny, calculated as an average of the expected polymorphic nucleotide proportions ± SD, are shown for each combination. The top panels show recombination breakpoints, identified using the LUMPY package, and their proportions in relation to the NGS read coverage. Only the recombination sites with more than 10 supporting events exceeding 0.02% of the NGS coverage are shown; black and red diamonds show the recombination sites (black for the predominant isolate at 5′, red for the predominant isolate at 3′). In the lower panels, the orange bars show ratios between the expected polymorphic nucleotides for each pairwise combination along with trend lines. The black bars show the ratios of the second most abundant nucleotide to the total coverage at the positions, which were not polymorphic for a given genotype pair, indicative of the background wild-type DWV infection. For each graph, x-axes show nucleotide position in the DWV genome and y-axes show recombinant read proportions (top panes) or polymorphic nucleotide ratios (bottom panes). dpi, days postinoculation; DWV, deformed wing virus; NGS, next-generation sequencing. (TIFF) [file pbio.3000502.s004.tiff]

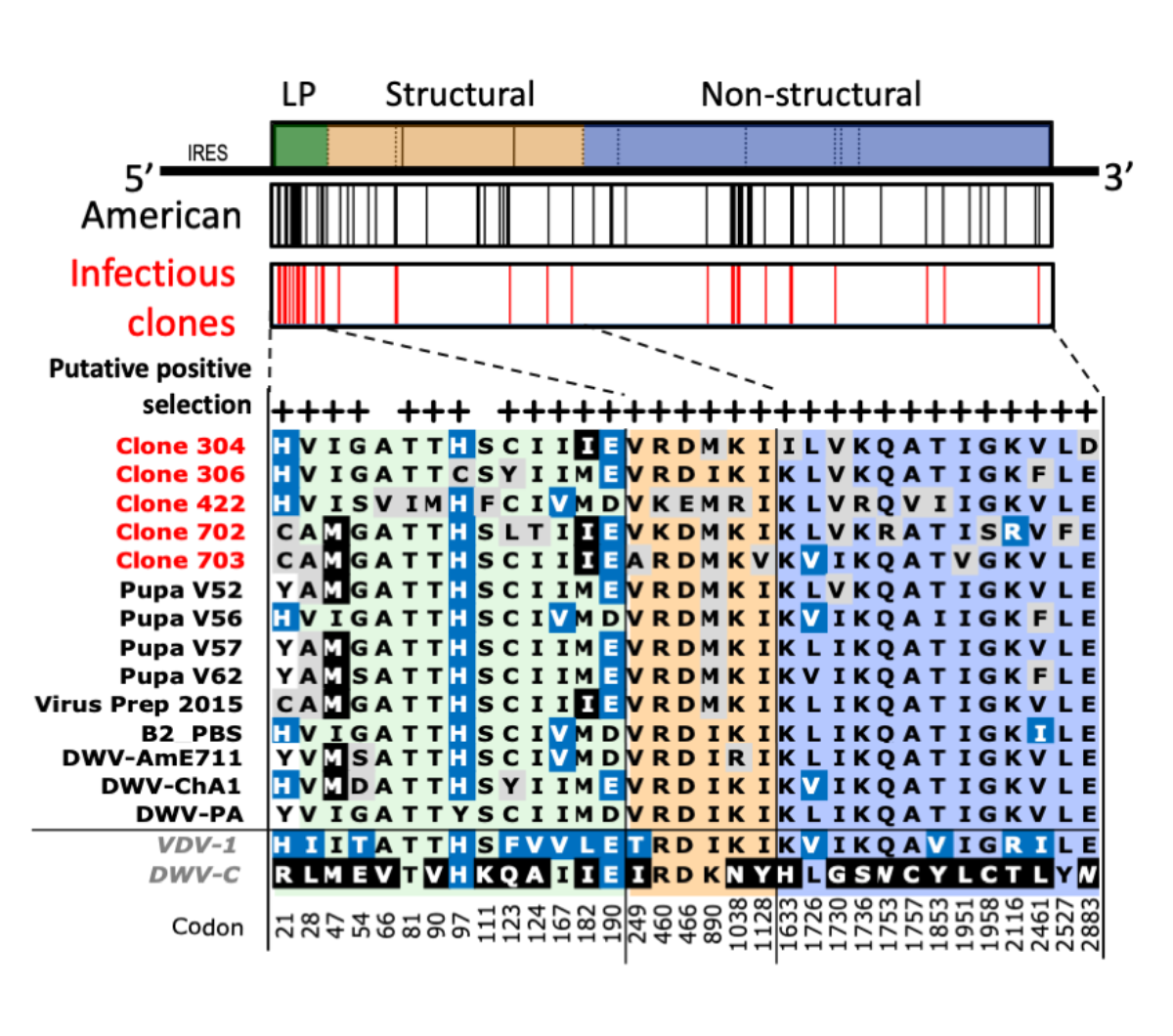

Supplement: S5 Fig — Alignment shows divergent positions in the full-length North and South American DWV sequences and in the infectious US DWV cDNA clones. Codon numbers are indicated below. Positions marked with “+” correspond to putative codons under positive diversifying selection in DWV strain from Pennsylvania (AY292384), which is used as the reference genotype. DWV, deformed wing virus. (TIFF) [file pbio.3000502.s005.tiff]

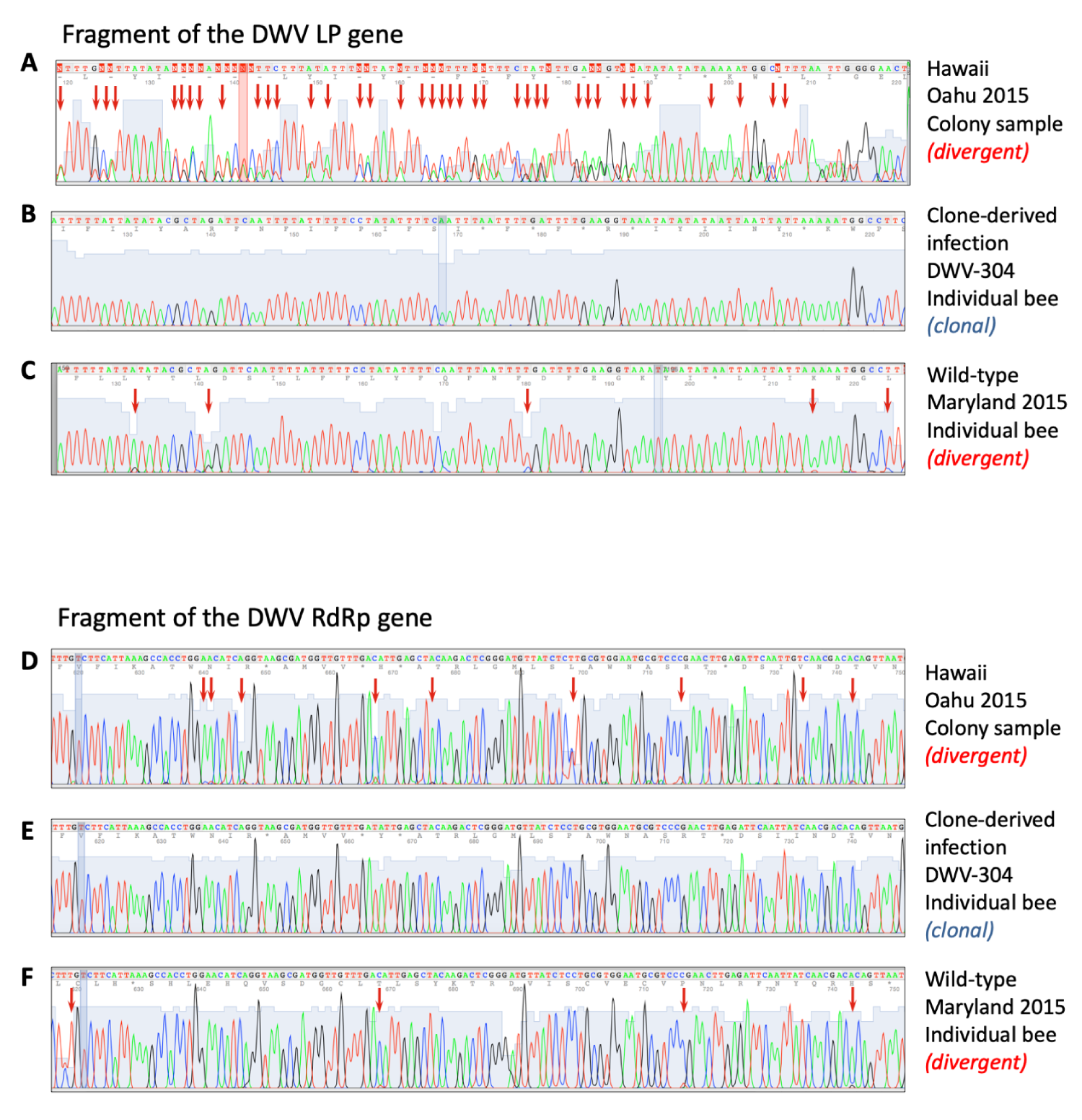

Supplement: S6 Fig — Electropherograms of the direct sequencing of RT-PCR fragments corresponding to sections of DWV RNA: (A-C) LP region (positions 1,045–1,158) and (D-F) RdRpol fragment (positions 8,645–8,744, sequenced in the Hawaiian DWV diversity study to demonstrate DWV clonality in Oahu Island DWV in 2009 [9]). The fragments were amplified using RNA extracted from (A, D) pooled honey bees collected from a Varroa-infested apiary on Oahu Island, Hawaii, in 2015; (B, E) an individual honey bee pupa injected with clone-derived DWV-304; (C, F) an individual honey bee pupa infected with Maryland wild-type DWV (2015 sample source). Divergent positions are indicated with arrows. DWV, deformed wing virus; LP, leader protein; RdRpol, RNA-dependent RNA polymerase; RT-PCR, reverse-transcription PCR. (TIFF) [file pbio.3000502.s006.tiff]

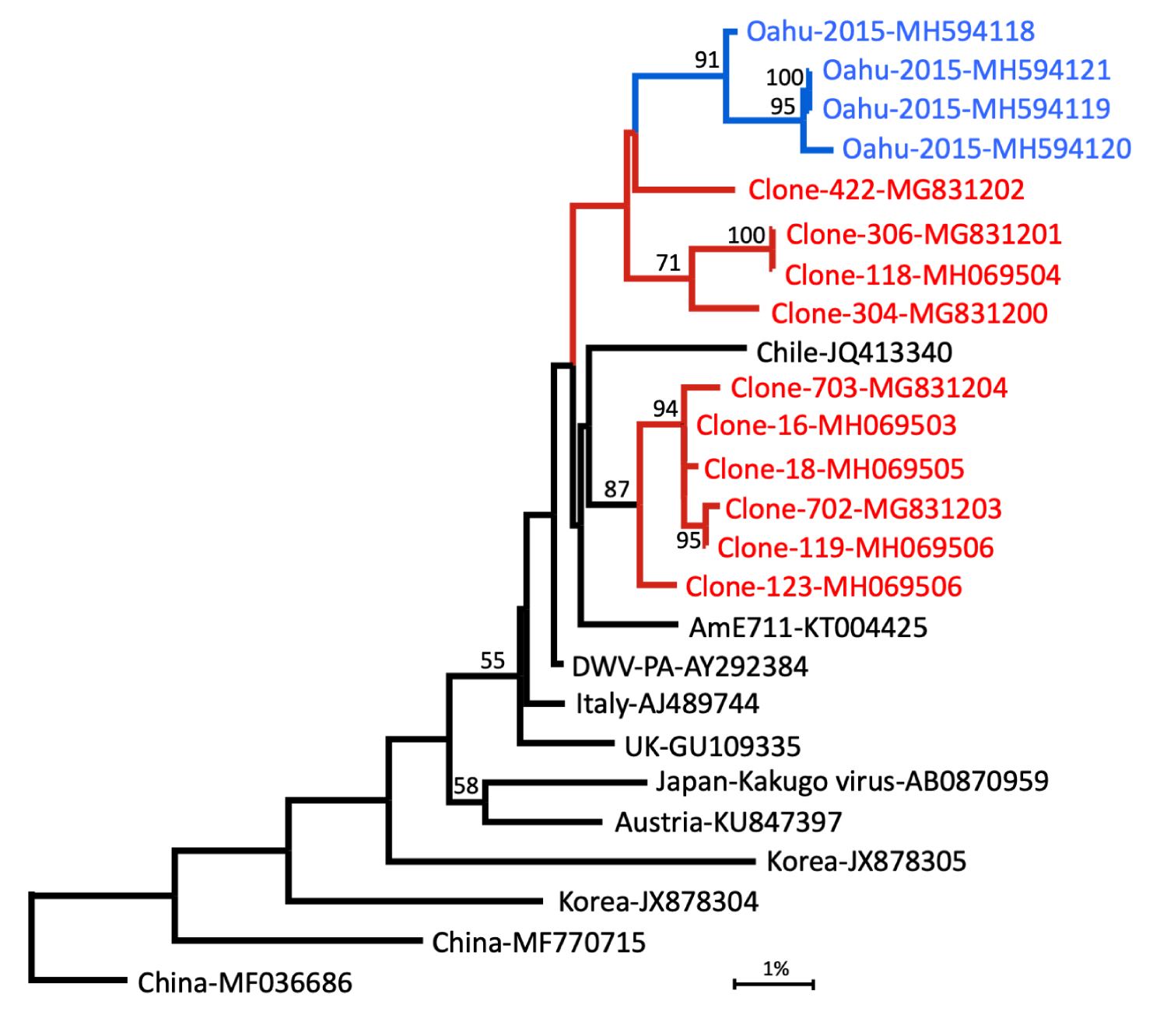

Supplement: S7 Fig — Maximum likelihood phylogenetic tree corresponds to the section of the DWV genome coding for the LP and adjacent parts of 5′ IRES and the structural gene block (positions 922–2,048). Blue nodes connect the cloned sequences from a Hawaiian apiary (Oahu Island) sampled in 2015; red nodes connect the cloned sequences and the full-length DWV genomes from a Maryland Varroa-infested colony sampled in 2015. Bootstrap values for 1,000 replicates are shown for the groups with more than 50% bootstrap support. Scale bar shows genetic distance (%). Isolates are labeled with their clone identifier or country of origin as well as their NCBI accession. DWV, deformed wing virus; IRES, internal ribosome entry site; LP, leader protein; NCBI, The National Center for Biotechnology Information. (TIFF) [file pbio.3000502.s007.tiff]
