# Supplementary material for: Dynamic evolution in the key honey bee pathogen deformed wing virus: Novel insights into virulence and competition using reverse genetics
Source: PLoS Biol. 2019 Oct 10;17(10):e3000502. doi: 10.1371/journal.pbio.3000502 (PMC6805011; doi:10.1371/journal.pbio.3000502)
Supplement: S1 Text — Nucleotide alignment of the 283-nt section of DWV isolates DWV-304 and DWV-422 (GenBank accession numbers MG831200 and MG83120), positions 1,242–1,524 in the DWV genomic RNA, which were used to generate dsRNA. dsRNA, double-stranded RNA; DWV, deformed wing virus. (PDF) [file pbio.3000502.s008.pdf]

S1 Text 1. Double-stranded (ds) RNAs specific to the cloned DWV genotypes.
Nucleotide alignment of the 283 nt section of DWV isolates DWV-304 and DWV-422, (Genbank accession numbers MG831200 and MG83120), positions 1242-1524 in the DWV genomic RNA, which were used to generate dsRNA.

```
ds304      GGGAAGTTGATGAAGCCAGGCGGCGCCGAGTCATCAAACGTTTGGCGCTGGAGCAAGAAC   60
ds422      GGGAAGTTGATGAAGCCAGGCGGCGCCGAGTCATTAAACGTTTGGCGCTAGAGCAAGAAC   60
           ********************************* *************** **********

ds304      GTATTCGCAACGTTCTTGACGCCGGCGTCTATGACCAGGCGACATGGGAACAGGAGGACG   120
ds422      GTATTCGTAACGTTCTTGACGCTAGCGTCTATGACCAGGCGACATGGGAACAGGAGGACG   120
           ******* **************  ************************************

ds304      CGCGCGATAATGAGTTCCTAACGGAACAATTAAACAATTTATATACTATTTATTCGATCG   180
ds422      TGCGCGATAATGAGTTCCTAACGGAACAATTAAATAATTTATATATTATTTATTCGATCG   180
            ********************************* ********** **************

ds304      CCGAACGTTGCACGCGTCGACCTATTAAAGAGCACTCTCCTATATCAGTTTCGAATAGGT   240
ds422      CTGAACGTTGTATGCGTCGGCCTATTAAAGAGCACTCTCCTATATCAGTTTCGAATAGGT   240
           * ******** * ****** ****************************************

ds304      TTGCTCCATTGGAATCCCTCAAAGTCGAGGTCGGTCAAGAAGC             283
ds422      TTGCTCCACTGGAATTCCTCAAGGTCGAGGTCGGTCAAGAAGC             283
           ******** ****** ****** ********************
```
